# Treatment options applied to the preclinical studies using animal models for Chagas Disease: a systematic review and meta-analysis

**DOI:** 10.12688/f1000research.150723.3

**Published:** 2025-05-16

**Authors:** Laura Yesenia Machaca-Luque, Mayron Antonio Candia-Puma, Brychs Milagros Roque-Pumahuanca, Haruna Luz Barazorda-Ccahuana, Luis Daniel Goyzueta-Mamani, Alexsandro Sobreira Galdino, Ricardo Andrez Machado-de-Ávila, Rodolfo Cordeiro Cordeiro Giunchetti, Eduardo Antonio Ferraz Coelho, Miguel Angel Chavez-Fumagalli

**Affiliations:** 1 Facultad de Ciencias Farmacéuticas, Bioquímicas y Biotecnológicas, Universidad Católica de Santa María, Arequipa, 04000, Peru; 2 Computational Biology and Chemistry Research Group, Vicerrectorado de Investigación, Universidad Católica de Santa María, Pedro Vilcapaza, Arequipa, 04000, Peru; 3 Laboratório de Biotecnologia de Microrganismos, , MG e Instituto Nacional de Ciência e Tecnologia em Biotecnologia Industrial, Universidade Federal de Sao Joao del-Rei, Divinópolis, State of Minas Gerais, 35501-296, Brazil; 4 Programa de Pós-Graduação em Ciências da Saúde, University of the Extreme South of Santa Catarina, Criciúma, State of Santa Catarina, 88806-000, Brazil; 5 INCT-DT, Instituto Nacional de Ciencia e Tecnologia em Doencas Tropicais, Salvador, State of Bahia, 40015-970, Brazil; 6 Laboratório de Biologia das Interações Celulares, Instituto de Ciências Biológicas, Universidade Federal de Minas Gerais, Belo Horizonte, State of Minas Gerais, 31270-901, Brazil; 7 Programa de Pós-Graduação em Ciências da Saúde: Infectologia e Medicina Tropical, Faculdade de Medicina,, Universidade Federal de Minas Gerais, Belo Horizonte, State of Minas Gerais, 31270-901, Brazil

**Keywords:** Chagas disease, treatment, efficacy, systematic review, meta-analysis, parasitemia, preclinical studies

## Abstract

**Background:**

Chagas disease (CD) is a neglected tropical disease endemic to Latin America, has emerged as a global health concern due to the migration of infected individuals. With its epidemiological complexity, by difficulty to obtain appropriate diagnoses and poor treatment, the search for novel therapeutic options remains.

**Methods:**

In this context, we conducted a systematic review and meta-analysis of preclinical studies employing animal models to verify the progress in CD treatment. We searched the PubMed database for CD treatment studies published between 1990 and 2023, adhering to the PRISMA guidelines.

**Results:**

Twelve papers met the inclusion criteria. The findings indicate that the fifteen treatment alternatives examined, mainly between 2010 and 2014, demonstrated efficacy in experimental CD models, evidenced by significant parasitemia reduction. Bis-triazole DO870 and VNI were effective in the acute and chronic phases, respectively. However, of these emerging therapies, only posaconazole and fexinidazole have progressed to clinical trials, yielding unsatisfactory outcomes as CD monotherapies

**Conclusions:**

This meta-analysis highlights the existence of promising new drug candidates for CD treatment, but most remain in the preclinical stages. Those that reached clinical trials did not demonstrate optimal results, underscoring the ongoing challenges in CD therapy. Collaborative efforts among the academic community, pharmaceutical industries, funding agencies, and government agencies are urgently needed to accelerate the development of more effective medications against CD.

**Inplasy registration:**

INPLASY202430101 (25/03/2024)

**Abbreviations**
CD: Chagas disease
CI: Confidence interval
c _neg_
: Control group negative
c _pos_
: Control group positive
DNA: Deoxyribonucleic acid
I ^2^
: I-squared
INPLASY: International Platform of Registered Systematic Review and Meta-analysis Protocols
MeSH: Medical Subject Headings
PRISMA: Preferred Reporting Items for Systematic Reviews and Meta-Analyses
Q: Q value
RR: Risk Ratio
T ^2^
: tau-squared
t _neg_
: Treated group negative
t _pos_
: Treated group positive
*T. cruzi*
: *Trypanosoma cruzi*
WHO: World Health Organization

## Introduction

Chagas Disease, caused by the protozoan parasite *Trypanosoma cruzi*, is a neglected tropical illness endemic to Latin America, predominantly impacting rural and impoverished communities. ^1^ Worldwide, the disease affects an estimated 6 to 7 million people, with 10,000 deaths each year. ^2^ Additionally, there are emerging reports indicating the spread of Chagas Disease to non-endemic countries, fueled by factors such as human migration, travel patterns, globalization, and climate change, thereby introducing new complexities to disease management and surveillance efforts. ^3^ The disease presents two distinct clinic phases: acute and chronic. Acute CD is often characterized by nonspecific symptoms such as fever, malaise, and local edema at the site of parasite entry, ^4^ while chronic infection potentially lead to serious cardiac and gastrointestinal problems such as megaesophagus, megacolon, arrhythmias, and cardiomyopathy. All of these complications further increase the risk of sickness and death. ^5^ The pathogenesis of Chagas disease is a complex process influenced by various factors, including transmission dynamics and environmental conditions. Effective disease control necessitates addressing factors such as poor sanitation, shared housing, and “epidemiological blind spots” that hinder surveillance and treatment strategies. ^6^^,^^7^

The current therapeutic options for CD mostly consist of two nitroheterocyclic compounds, such as benznidazole and nifurtimox, developed in the late 1960s and early 1970s, respectively. ^8^^,^^9^ While these treatments have been widely used for decades, they are associated with significant side effects and require prolonged treatment regimens, which can affect patient adherence. ^10^ Although traditionally considered to have limited efficacy in chronic CD, recent evidence suggests that treatment may still provide clinical benefits, particularly in slowing disease progression. ^11^ Traditionally, it was thought that CD only became clinically evident after prolonged exposure to the parasite. However, it is now understood that clinical symptoms can be triggered by various factors, including host immune responses and parasite load, not merely by continued exposure. ^12^ Given this evolving perspective, the treatment of infected patients is now widely recommended unless specific contraindications exist. However, existing therapies still fail to achieve parasitological clearance in a substantial number of patients, and since sterilizing cure remains the primary therapeutic goal, the development of new drugs continues to be a priority. ^13^ ^,^ ^14^ The challenges in drug development include the complex biology of *T. cruzi* and host immune responses, as well as the lack of financial incentives for pharmaceutical companies to invest in treatments for a disease primarily affecting impoverished populations. ^15^ ^,^ ^16^

Recently, fundamental research, particularly in the preclinical stage, has been the focal point of efforts aimed at developing new treatment strategies for CD. These endeavors predominantly utilize techniques derived from in vitro or animal investigations. ^9^^,^^17^ Notably, animal model-based preclinical research has emerged as indispensable for evaluating novel CD treatment approaches. ^18^ Such studies facilitate the exploration of fundamental mechanisms of action and pharmacokinetic characteristics, while also providing valuable insights into the safety and efficacy of innovative therapies. ^19^ An additional challenge in evaluating treatment outcomes in clinical trials is the absence of a reliable cure biomarker, making it difficult to assess treatment efficacy accurately. This limitation affects the design and interpretation of clinical studies. ^20^

This systematic review and meta-analysis aim to evaluate the efficacy of different treatment options for Chagas disease in preclinical studies using animal models. The study compares the effectiveness of various anti-parasitic compounds in reducing parasitemia and improving disease outcomes, providing a quantitative assessment to identify promising candidates for future research. Given the heterogeneity in therapeutic approaches, we aim to answer the following research question: What is the efficacy of different treatment options in reducing parasitemia in preclinical models of Chagas disease, and how do they compare in terms of therapeutic outcomes?

## Methods

### Study protocol

This systematic review conducted following the Preferred Reporting Items for Systematic Reviews and Meta-Analyses (PRISMA) statement (Table S1). ^21^ With registration number INPLASY202430101 and DOI: 10.37766/inplasy2024.3.0101, the protocol for this systematic review was registered on the International Platform of Registered Systematic Review and Meta-analysis Protocols (INPLASY) website. The entire protocol is accessible at inplasy.com ( https://inplasy.com/inplasy-2024-3-0101/).

### Information sources and search strategy

The literature was searched for phrases about CD therapy using the MeSH (Medical Subject Headings) term “Chagas Disease”. Using the VOSviewer program (version 1.6.20), the findings were shown in a network diagram showing the co-occurrence of MeSH keywords. ^22^ We looked at clusters in the network map to choose phrases associated with CD therapy. Furthermore, a second round of searches was carried out by linking each MeSH term identified in the cluster analysis with the MeSH terms “Chagas Disease” and “Treatment Outcome”, which relate to assessing the outcomes of interventions used to combat diseases and determining their efficacy, ^23^ For the years 1990–2023, records were obtained from the bibliographic database PubMed ( https://pubmed.ncbi.nlm.nih.gov/, last accessed 24 May 2023).

### Selection criteria and data extraction

The procedure for selecting studies for this review comprised three separate phases. During the initial identification phase, only animal studies published between 1990 and 2023 were taken into account. Duplicate articles, non-English publications, reviews, and meta-analyses were excluded at this stage. The subsequent screening phase involved checking the titles and abstracts of the identified articles, and in the eligibility/qualification phase, full-text studies highly relevant to the research question were retrieved, specifically focusing on treatment options for CD. Data on the type of compound used for treatment, dosage, duration of treatment, total sample size, number and species of experimental animals infected *with T. cruzi*, phase of CD, *T. cruzi* strain, sample type, and description of the controls were extracted from each of the chosen studies. Studies with insufficient data were excluded. In contrast, those that assessed the effectiveness of therapy by disclosing parasitemia data were retained, considering a 70% decrease in parasitemia as a positive treatment for both the treated and control groups. Options for treatment that included a drug in addition to a conventional CD medication, like nifurtimox or benznidazole, weren’t included. Drugs used in monotherapy were emphasized to confirm which ones show higher effectiveness. WebPlotDigitizer was used for data analysis when the information was shown graphically. ^24^^,^^25^ WebPlotDigitizer version 5 is a semi-automatic tool that lets you manually plot two-dimensional charts and extract numerical data. It is free online ( https://automeris.io/WebPlotDigitizer/) or as desktop software that may be downloaded. ^24^ This instrument has been employed in several systematic reviews and meta-analyses, encompassing therapy efficacy assessment. ^26^^–^^28^ L.Y.M.-L. carried out the data extraction, and M.A.C.-P. Independently checked it. If there were any differences, M.A.C.-F. was consulted and discussed.

### Quality assessment and risk of bias

The methodological quality and risk of bias of the included animal studies were assessed using SYRCLE’s risk of bias tool, ^29^ which evaluates key domains such as selection bias, performance bias, detection bias, attrition bias, reporting bias, and other biases specific to animal studies. Selection bias examines random allocation, performance bias ensures uniform treatment, detection bias checks for blinding, attrition bias looks at dropout management, and reporting bias ensures complete reporting of outcomes. Additional biases specific to animal studies are also considered. To evaluate the overall quality of evidence, the GRADE approach ^30^ was applied, which assesses certainty based on risk of bias, inconsistency, indirectness, imprecision, and publication bias, categorizing evidence into four levels: high, moderate, low, or very low certainty.

### Statistical analysis

Results were entered into a Microsoft Excel (version 2108, Microsoft Corporation, Redmond, WA, USA) spreadsheet and analyzed in the R programming environment (version 4.2.3) using the “metafor” package https://www.metafor-project.org/doku.php/metafor (accessed on 21 February 2024). ^31^ Plotting the synthesis findings and estimating a random-effects model are just a few of the numerous tasks that may be performed by the user with the help of the “metafor” package. ^31^^,^^32^ The number of treated animals who tested positive or negative for CD (tpos and tneg, respectively), and the corresponding number of untreated animals (control) who tested positive or negative for CD (cpos and cneg, respectively), were evaluated independently for each therapeutic option available with the “metafor” package”.

A Random Effects Model (RE model) was utilized in the meta-analysis process. According to this model, every study has a unique true effect that varies depending on the differences in animals, interventions, or circumstances between studies. ^33^ The Q value (Q), the I-squared (I ^2^), the tau-squared (T ^2^), and the risk ratio (RR) were also computed. One indicator of heterogeneity among the studies in the meta-analysis is the Q. The sum of the squares representing the discrepancies between the weighted overall effect and the observed effects are used to compute it. A high Q score indicates higher levels of study heterogeneity. ^34^

The overall percentage of variance in the estimated effects that results from heterogeneity between studies as opposed to random variation is measured by the I ^2^ statistic. It is represented as a percentage and computed as (Q - df ) /Q. ^35^ Excessive I ^2^ values (above 50%) suggest significant variability among the studies. ^36^ In a random effects model, the variation between studies is represented by the T ^2^. A random-effects model was chosen over a fixed-effects model for several reasons. First, this model accounts for variability in true effects across studies, which is particularly relevant in preclinical research involving different animal models, experimental conditions, and treatment protocols. Even if a low I ^2^ suggests minimal observed heterogeneity, unmeasured differences in study design, strain susceptibility, infection models, or pharmacokinetic responses may still exist. ^37^ Second, the T ^2^ statistic estimates the variance of true effect sizes beyond sampling error, allowing for uncertainty in underlying biological mechanisms affecting treatment efficacy. ^33^ Finally, since preclinical studies often involve small sample sizes and diverse methodologies, a more conservative approach was adopted with the random-effects model, which provides more generalizable estimates by assuming a distribution of possible effects rather than a single fixed effect size. ^38^ As the RR of the event happening in the exposed or treated group compared to the unexposed or untreated group, the RR measures the connection between an exposure or treatment and an outcome of interest. ^39^ Compared to the reference group, an exposure or treatment is linked to a higher outcome risk if the relative risk (RR) is more significant than 1. Conversely, a lower risk is suggested when the RR is less than 1. If the relative risk (RR) is 1, then there is no variation in risk among the groups. Confidence intervals are a useful tool for assessing the accuracy of the estimate, in addition to the RR value. ^40^ In this study, the event considered for RR calculation is the negativization of parasitemia (TC-) at the end of the study. Therefore, an RR > 1 indicates a higher probability of parasite clearance in the treated group compared to the control group, reflecting greater treatment efficacy in reducing parasite burden. For all computations, a 99% confidence level was used, with a 0.1 continuity adjustment applied as necessary.

## Results

### Data sources and study selection

**Figure 1.**
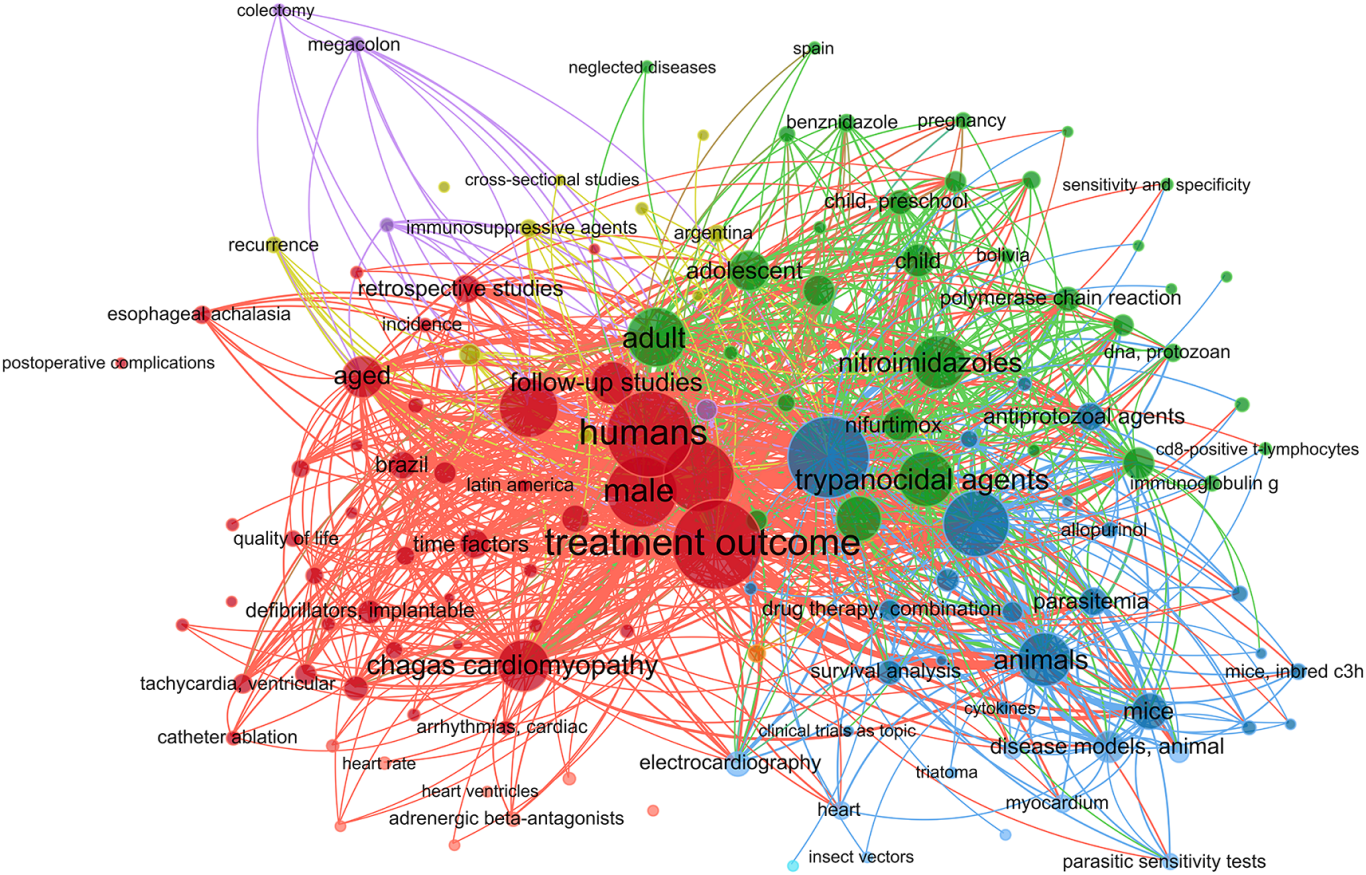
A network diagram using VOSviewer and PubMed represented CD treatment results based on MeSH term.

In this work, we performed a systematic review and meta-analysis to evaluate the effectiveness of many treatment options for lowering parasitemia. We considered both established therapies and recently developed, potentially effective medicines for CD. Figure S1 shows a flowchart of the study approach. A search for the MeSH term “Chagas Disease” AND “Treatment Outcome” was performed in the PubMed database and a MeSH term co-occurrence network map was developed. Through the search, 323 scientific publications were published between 1990 and 2023. A network map with 1,020 keywords was produced, with the minimal number of keyword occurrences set at five ( Figure 1). Three major clusters were found to have formed during the network map study. Terms including “nitroimidazoles”, “nifurtimox”, “benznidazole”, and “antiprotozoal agents” were found in the cluster about the therapy of CD (highlighted in green). There were other similar denominators, including phrases like “treatment outcome,” “humans,” “Chagas disease,” “female,” “male,” and “ *Trypanosoma cruzi*” ( Figure 1).

The terms found in the first analysis were used to perform a second search in the PubMed database. After the new phrases were associated with “Chagas Disease” and “Treatment Outcome”, the following new search strings were generated: (Chagas Disease [MeSH Terms]) AND (Treatment Outcome [MeSH Terms]) AND (nifurtimox [MeSH Terms]), (Chagas Disease [MeSH Terms]) AND (Treatment Outcome [MeSH Terms]) AND (nitroimidazoles [MeSH Terms]), and (Chagas Disease [MeSH Terms]) AND (Treatment Outcome [MeSH Terms]) AND (Therapeutics [MeSH Terms]) for commonly used treatments (nifurtimox and benznidazole) as well as recently developed treatments against CD.

**Figure 2.**
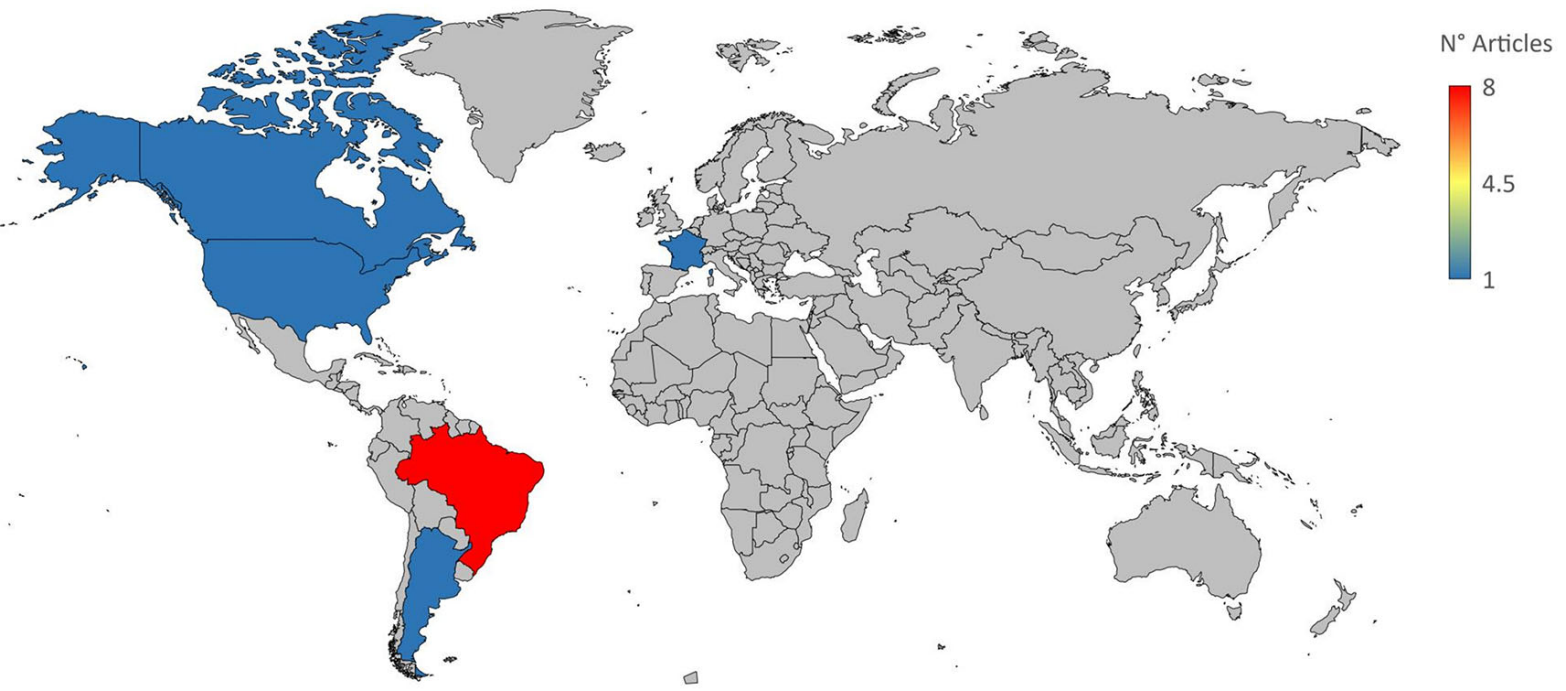
A demographic breakdown of international research on novel CD therapy options included in the meta-analysis.

There were 40, 107, and 112 studies chosen for the first, second, and third search strings, respectively. We excluded 91, 90, and 66 articles in the identification, screening, and eligibility phases, respectively, based on the three-step selection criteria that we employed. This resulted in the selection of 12 studies for the meta-analysis, one published in 2001, and the others between 2010 and 2014, several of which discussed various treatment choices; as a result, 30 publications in total were included in the investigation. In these investigations, the following novel chemical compounds were examined: Posaconazole, AmBisome ^®^, Cyclopalladated complex 7a, Fexinidazole, Psilostachyin A, Cynaropicrin, Reversible cruzipain inhibitors Cz007 and Cz008, dehydroepiandrosterone-sulfate, VNI, (−)−hinokinin-loaded microparticles, allopurinol, clomipramine, GW788388, and Bis-triazole D0870 ( Table 1). The most significant number of studies utilizing animal models to investigate potential novel treatments for CD have been conducted in Brazil. In addition, albeit to a lesser degree, studies on this subject have also been done in several other nations, including Argentina, Canada, France, and the United States ( Figure 2).

**Table 1.** Chemical compounds evaluated in preclinical studies using animal models of CD.

Reference	Molecule	Chemical Structure	Route, dose (mg/kg/day)
^41^^,^^42^	Posaconazole	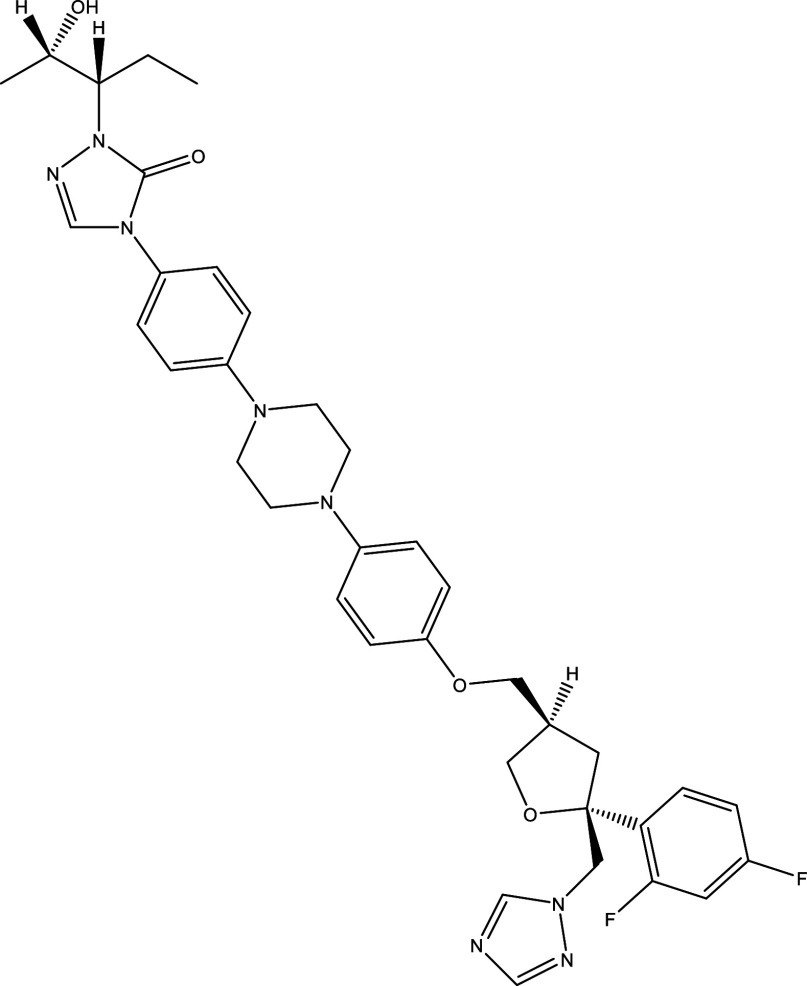	p.o., 20
^41^	AmBisome ^®^	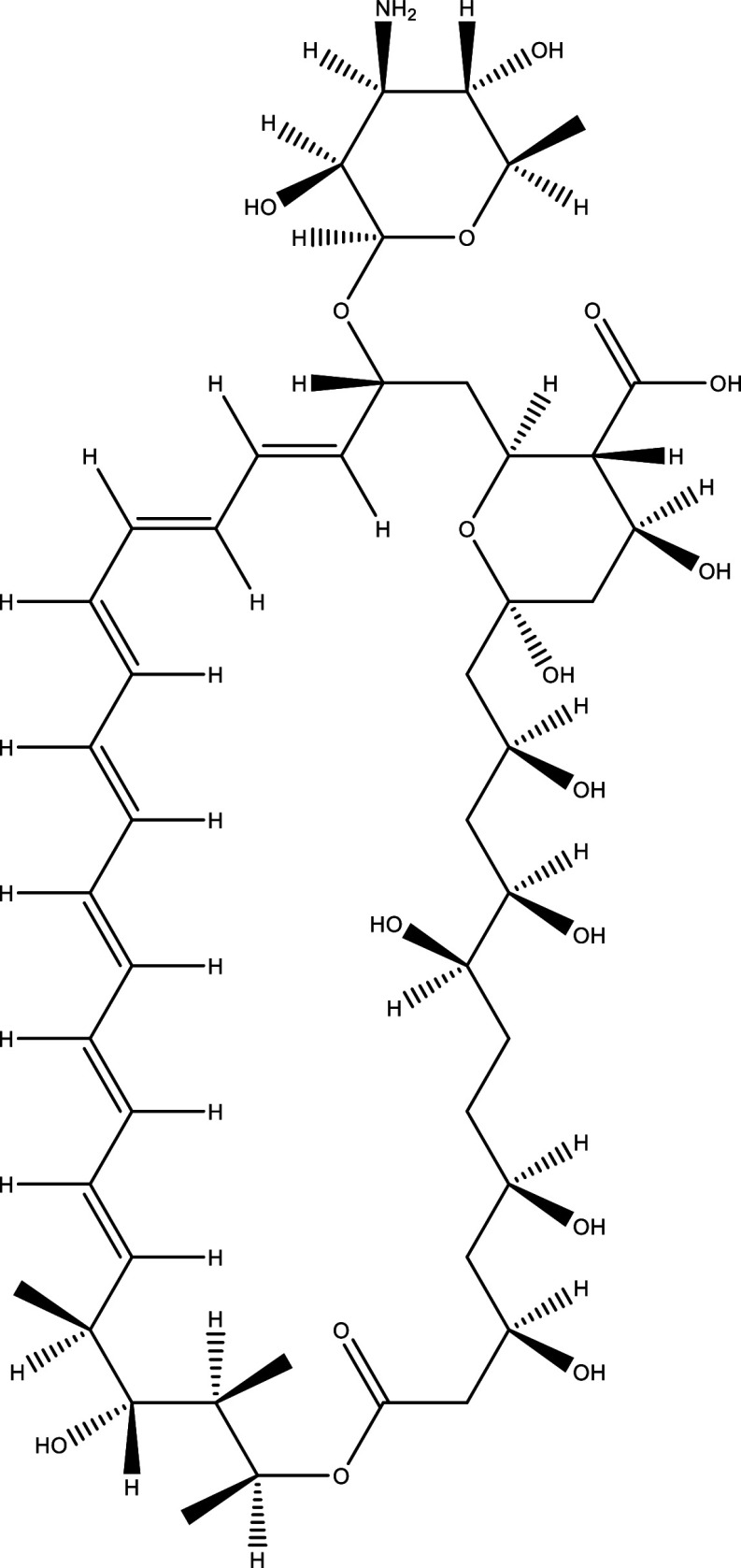	i.p., 25
^45^	Cyclopalladated complex 7a	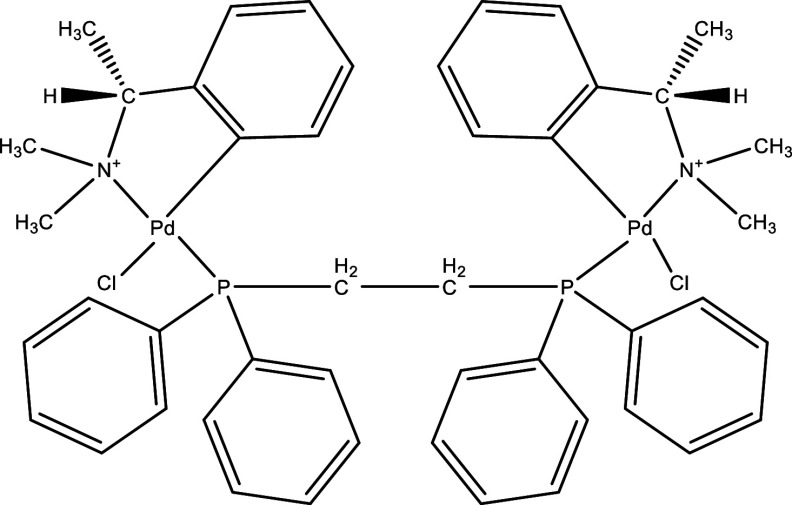	i.p., 0.12
^46^	Fexinidazole	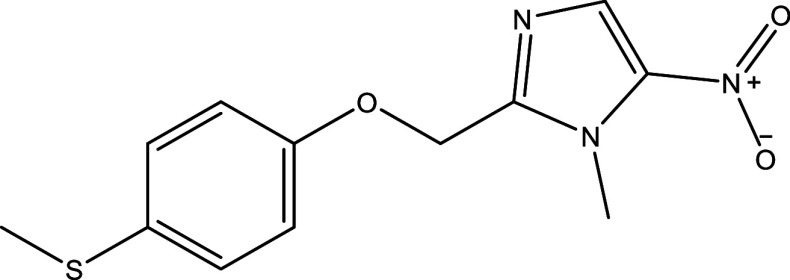	p.o., 300
^47^	Psilostachyin A	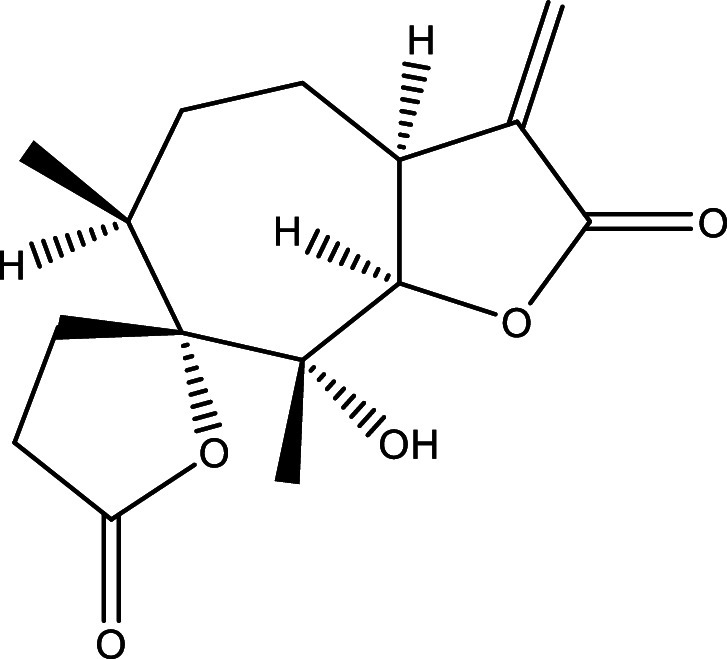	i.p., 50
^47^	Cynaropicrin	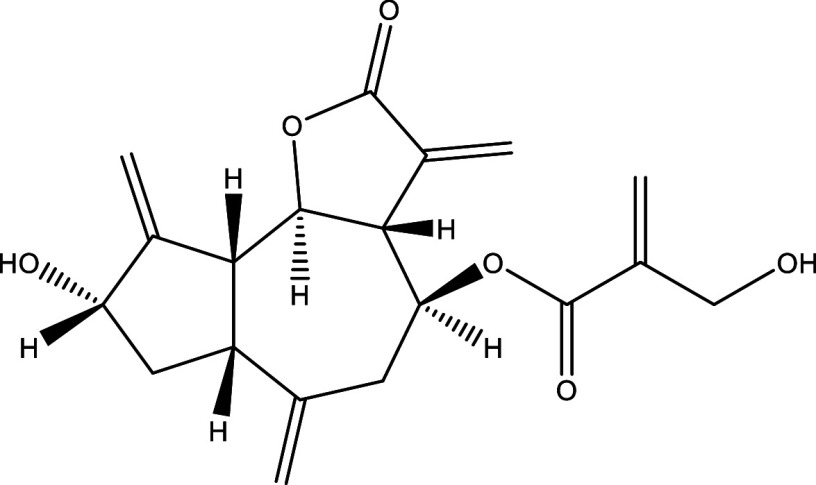	i.p., 50
^44^	Reversible cruzipain inhibitor Cz007	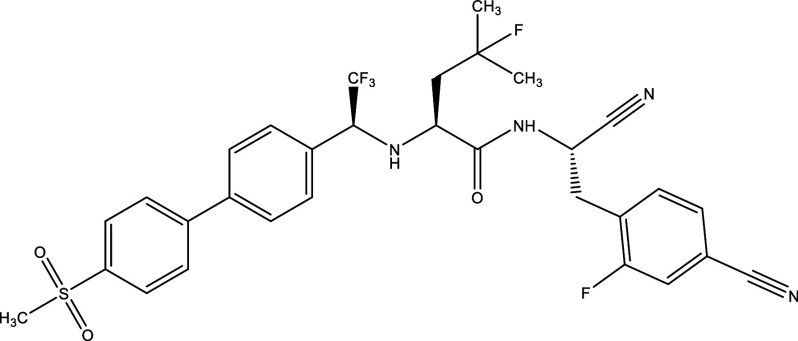	p.o., 50
^44^	Reversible cruzipain inhibitor Cz008	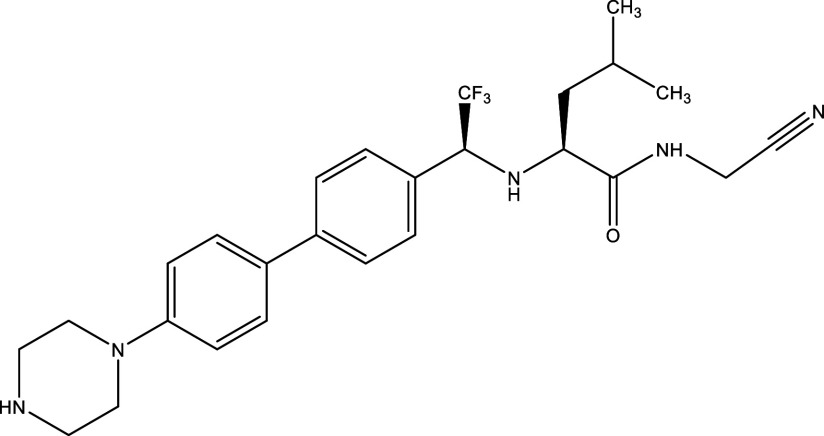	p.o., 50
^48^	Dehydroepiandrosterone-sulfate	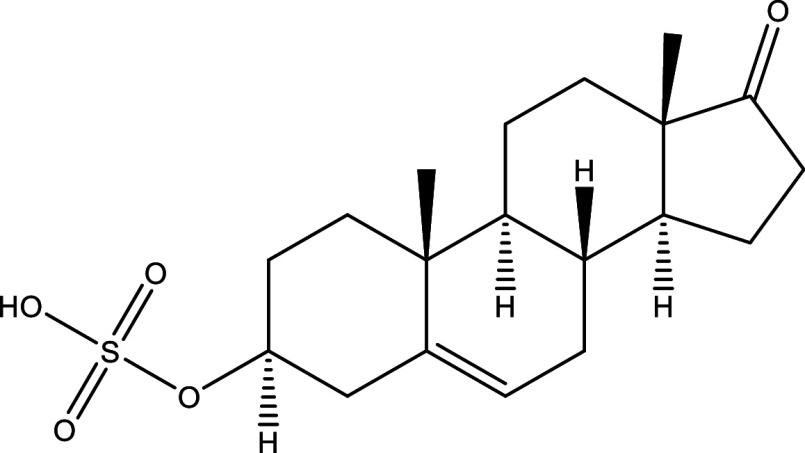	s.c., 40
^49^	VNI	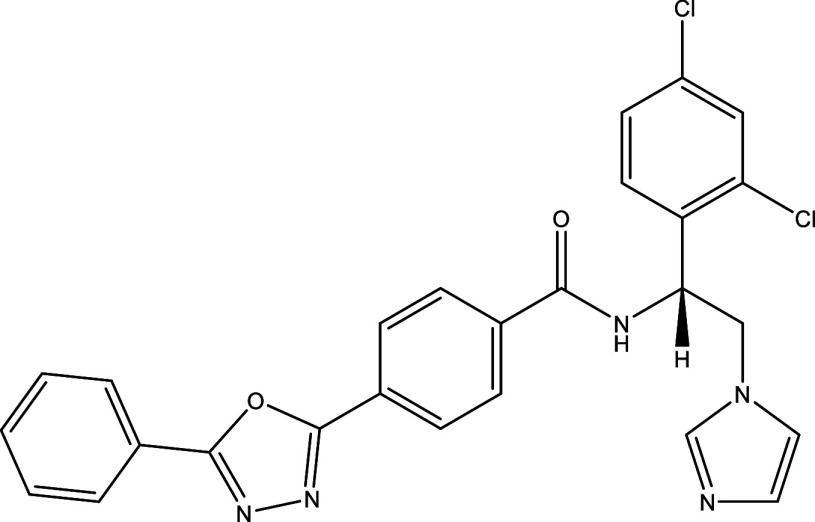	p.o., 50
^50^	(-)-Hinokinin	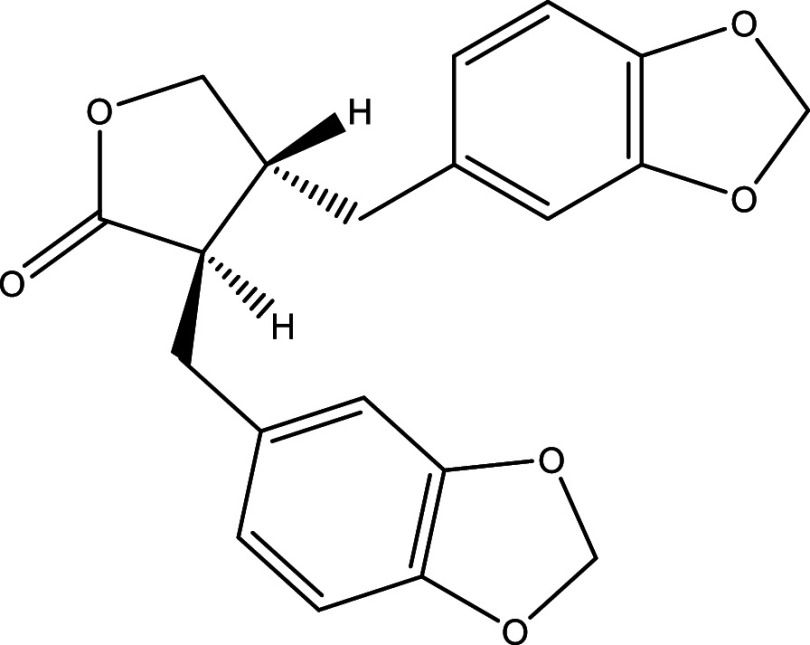	s.c., 20
^51^	Allopurinol	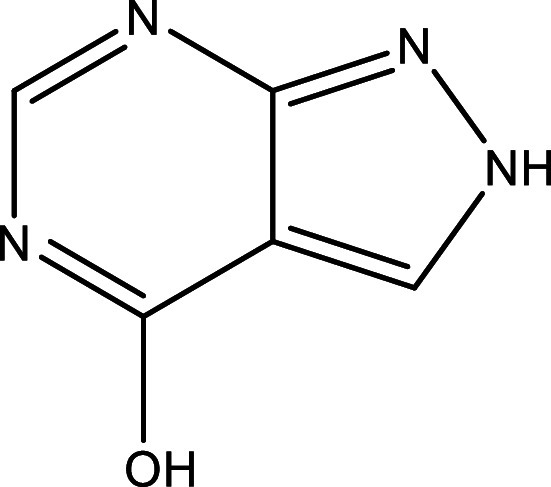	p.o., 15
^51^	Clomipramine	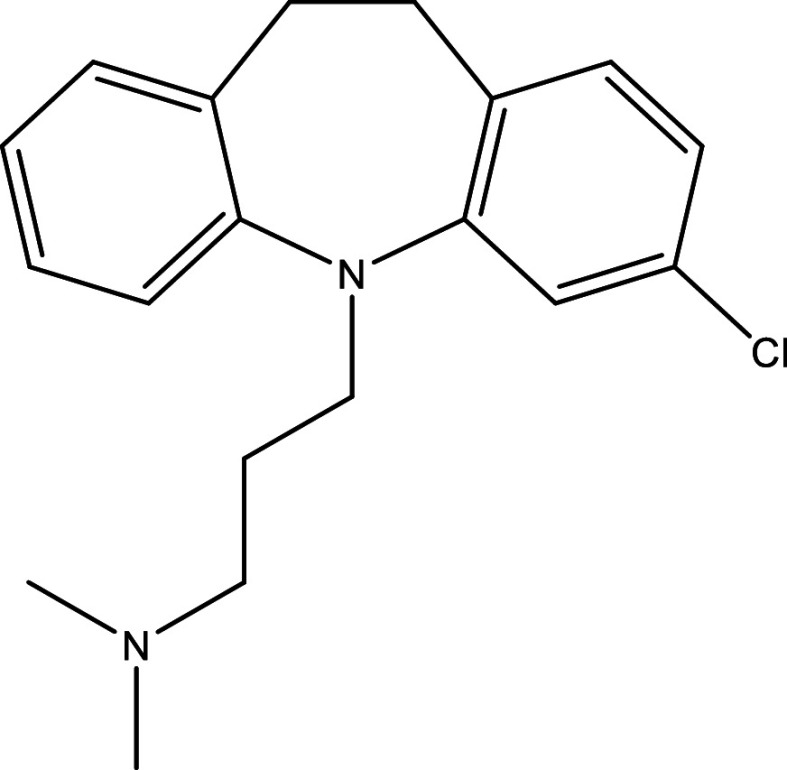	p.o., 5
^52^	GW788388	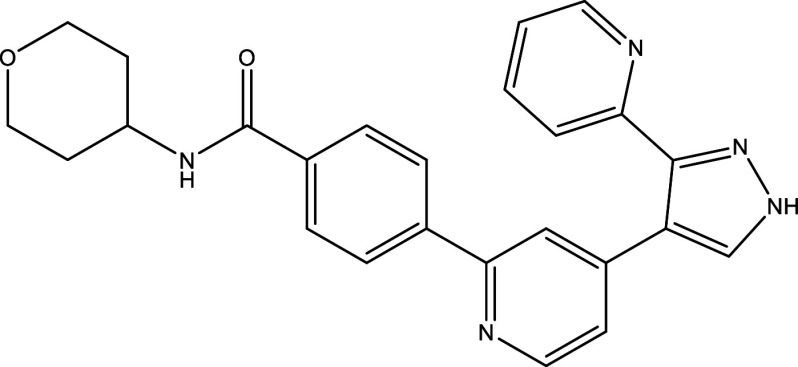	p.o., 3
^43^	Bis-Triazole DO870	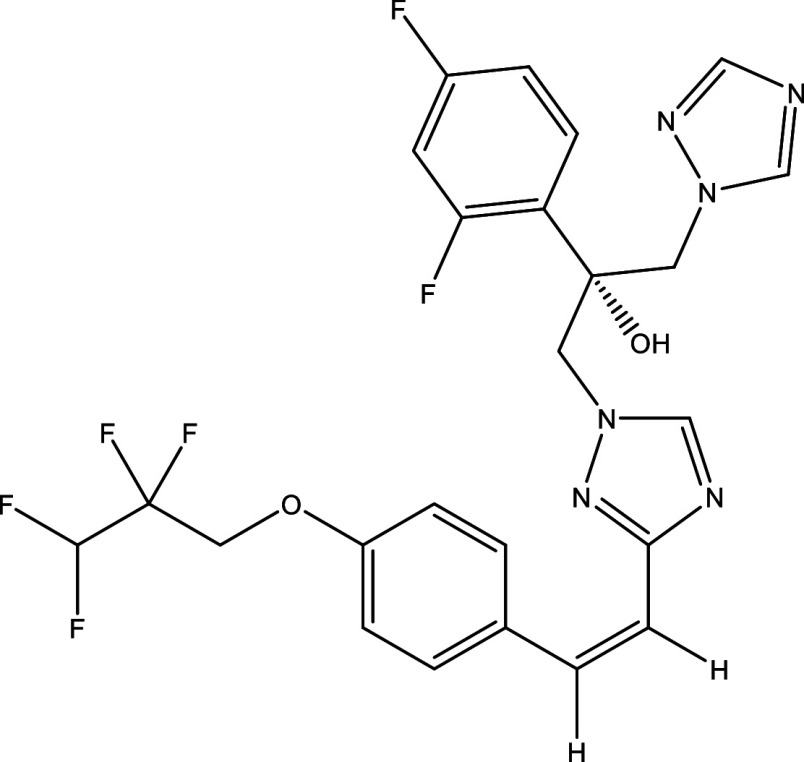	p.o., 5 i.v., 3

p.o. oral route, i.p. intraperitoneal route, s.c. subcutaneous route, i.v. intravenous route.

### Risk of bias and certainty of evidence

The risk of bias assessment using SYRCLE’s tool revealed a high overall risk across the included studies, with prevalent concerns regarding selection, performance, and detection biases. Issues such as random housing, allocation concealment, and incomplete reporting contributed to the high bias risk, primarily due to inadequate randomization, blinding procedures, and selective outcome reporting. These biases were common across the animal models used, raising concerns about the reliability of the findings. Despite some studies showing fewer issues, the overall risk of bias remained high, potentially affecting the interpretation of results (Table S2). The GRADE evaluation showed varying levels of evidence certainty. Several studies were rated with moderate certainty, suggesting promising efficacy of drug combinations and treatments in animal models of CD, but clinical validation in humans is necessary to confirm these findings. Some studies were rated with moderate-low certainty due to the lack of human validation, despite demonstrating superior effectiveness in preclinical settings. A few studies showed low certainty, especially when treatments exhibited limited or no efficacy. High certainty was attributed to studies demonstrating high efficacy in mice, suggesting potential for human application, though further validation is needed. Overall, the evidence was mixed, with promising results but significant gaps in clinical validation (Table S3).

### Meta-analysis of the treatment options for CD

**CD phase**

**Figure 3.**
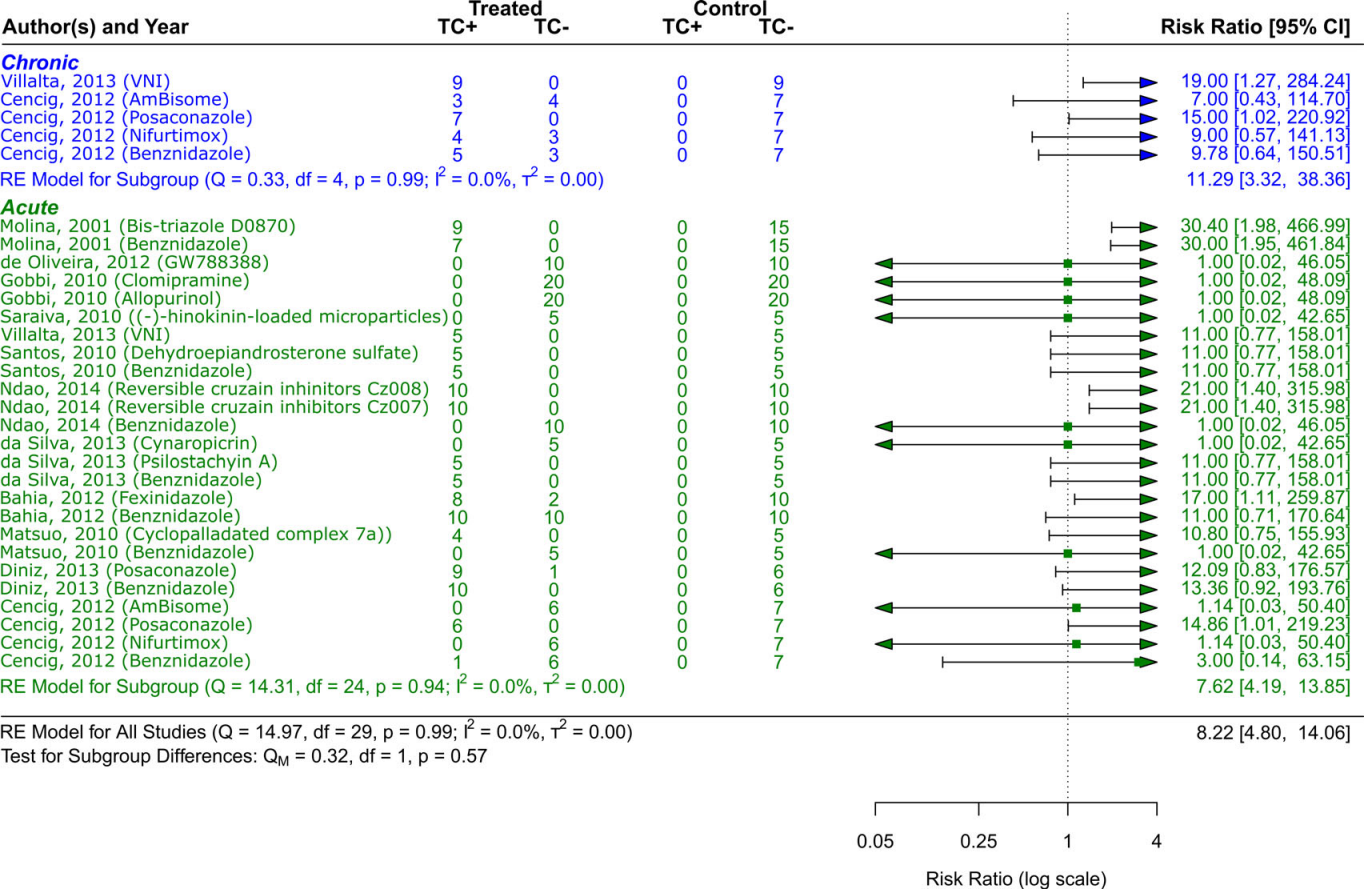
Forest plot comparing efficacy between treated and control groups according to CD phase. Error bars represent 95% CI. The square shapes represent the estimated RR. The vertical dashed line represents the no-effect line.

**Figure 4.**
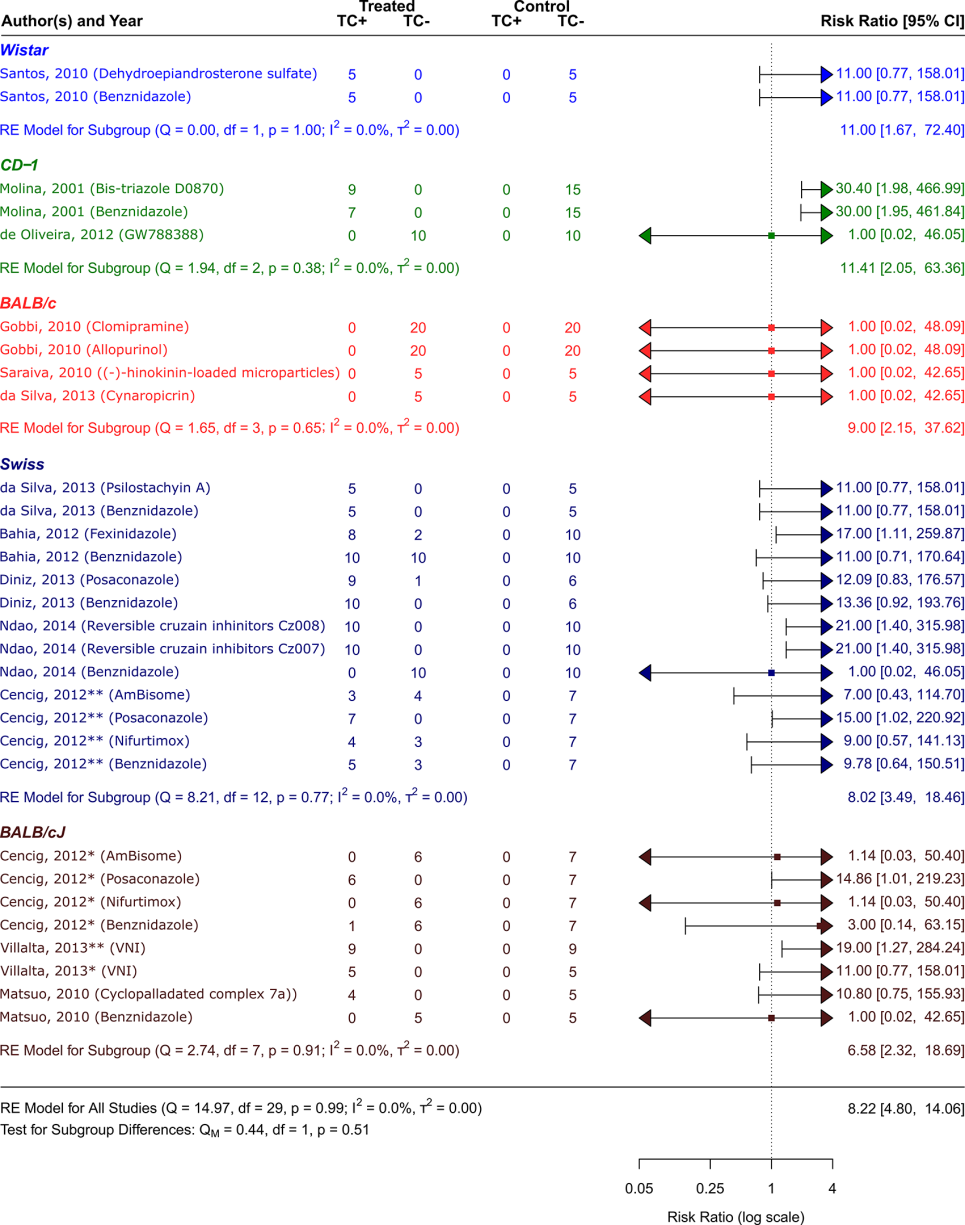
Forest plot comparing efficacy between treated and control groups according to animal strain. Symbol meaning: * Acute phase, ** Chronic phase. Error bars represent 95% CI. The square shapes represent the estimated RR. The vertical dashed line represents the no-effect line.

The acute phase of the disease and its treatment options posaconazole, AmBisome ^®^, Cyclopalladated complex 7a, Fexinidazole, Psilostachyin A, Cynaropicrin, Reversible cruzipain inhibitors Cz007 and Cz008, dehydroepiandrosterone-sulfate, VNI, (−)−hinokinin-loaded microparticles, allopurinol, clomipramine, GW788388, and Bis-triazole D0870 were also covered in twelve studies ^41^^–^^52^ that included a total of 741 animals. As Figure 3 shows, the treatment alternatives, particularly Bis-triazole D0870, proved to be effective compared to the control groups. (RR = 8.22, 95% CI [4.80, 14.06]). The tests showed that there was minimal heterogeneity (Q (df = 24) = 14.31, p = 0.94; I ^2^ = 0.0%; T ^2^ = 0.0%)). Two studies ^41^^,^^49^ with a total of 138 animals were examined that dealt with the chronic phase of the disease and its treatment choices (VNI, Benznidazole, Nifurtimox, Posaconazole AmBisome ^®^). The treatment alternatives were effective compared to the control groups, especially VNI, as Figure 4 illustrates (RR = 11.29, 95% CI [3.32, 38.36]). There was minimal heterogeneity, according to the tests (Q (df = 4) = 0.33, p = 0.99; I ^2^ = 0.0%; T ^2^ = 0.0%).

**Experimental animal models - strains**

**Figure 5.**
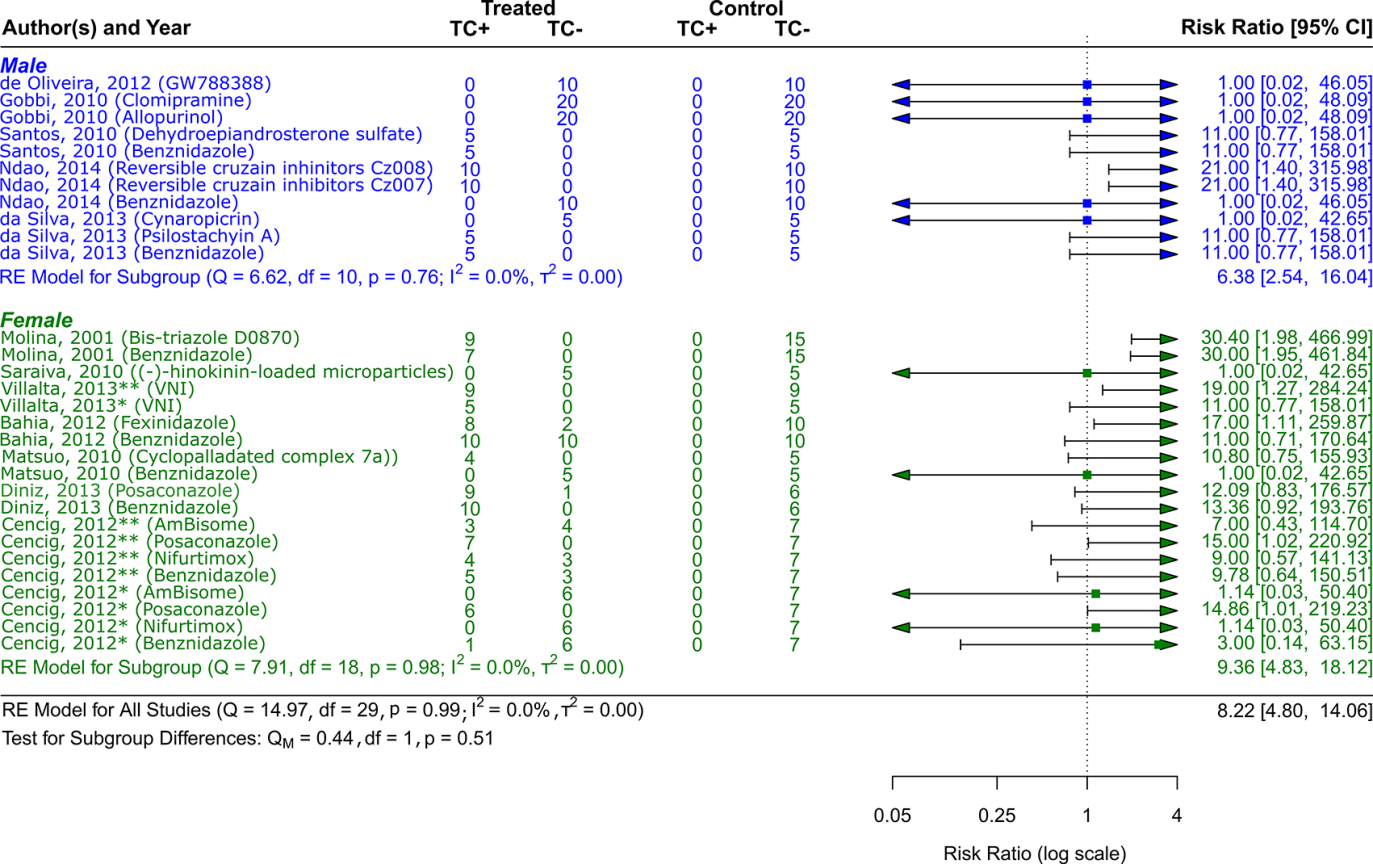
Forest plot comparing efficacy between treated and control groups by sex of animal models. Symbol meaning: * Acute phase, ** Chronic phase. Error bars represent 95% CI. The square shapes represent the estimated RR. The vertical dashed line represents the no-effect line.

The scientific articles were categorized into smaller groups based on the type of animal model that received a *T. cruzi* inoculation. Only one study ^48^ employed Wistar strain rats. Other mice strains were employed in the other studies: Swiss, ^41^^,^^42^^,^^44^^,^^46^^,^^47^ BALB/c, ^47^^,^^50^^,^^51^ CD-1, ^43^^,^^52^ and BALB/cJ. ^41^^,^^45^^,^^49^ As can be observed in Figure 5, CD−1 was the strain that responded to the treatment the best out of all the investigated strains. Its RR = 11.41, 95% CI [2.05, 63.36], indicates this. The tests showed that there was very little heterogeneity (Q (df = 2) = 1.94, p = 0.38; I2 = 0.0%; T2 = 0.0%)). On the other hand, the strain with the highest number of trials was the Swiss strain, which as shown in Figure 5 with an RR = 8.02, 95% CI [3.49, 18.46]) also showed a substantial response to the various treatment methods (Q (df = 12) = 8.21, p = 0.77; I ^2^ = 0.0%; T ^2^ = 0.0%) showed low heterogenicity. With an RR = 6.58, 95% CI [2.32, 18.69], and negligible heterogenicity (Q (df = 7) = 2.74, p = 0.91; I ^2^ = 0.0%; T ^2^ = 0.0%), BALB/cJ was the strain that responded to the treatment alternatives the least ( Figure 4).

**Experimental animal models - sex**

The sex of the animal model was used to categorize the scientific studies. RR = 9.36, 95% CI [4.83, 18.12] was the result of 7 studies including female experimental animals ^41^^–^^43^^,^^45^^,^^46^^,^^49^^,^^50^; Q (df = 18) = 7.91, p = 0.98; I ^2^ = 0.0%; T ^2^ = 0.0%, indicated a low level of heterogenicity ( Figure 5). The studies with the male sex of the experimental animals were 5, ^44^^,^^47^^,^^48^^,^^51^^,^^52^ showed a RR = 6.38, 95% CI [2.54, 16.04], with heterogenicity data of Q (df = 10) = 6.62, p = 0.76; I ^2^ = 0.0%; T ^2^ = 0.0% ( Figure 5). While analyzing the results, it is clear that female experimental animals are more susceptible than male counterparts to the various CD therapy approaches.

**Other research on CD treatments**

In the final analysis of the scientific publications, ^53^ one article about immunotherapy using DNA vaccines in mice was discovered. Immunotherapy boosts the body’s immune response, whereas conventional therapies utilize medications to treat a disease’s symptoms or underlying causes. ^54^ This study was left out of the meta-analysis because of their disparate biological objectives, which make them hard to evaluate in terms of efficacy and safety.

## Discussion

### Summary of main findings

Most of the studies included in the meta-analysis were conducted between 2010 and 2014. Progress in CD research has been slow due to multiple interrelated challenges. Scientifically, the biological complexity of *T. cruzi*—including its genetic diversity and ability to evade the host immune system—has hindered the identification of effective therapeutic targets. ^55^^–^^57^ Additionally, limited funding for CD-specific research has constrained large-scale clinical trials and resource availability, delaying therapeutic innovation. ^58^^–^^60^ The pharmaceutical industry’s lack of interest, driven by the low profitability of treatments for diseases prevalent in low-income regions, has further slowed progress. ^61^^,^^62^ The rise of drug-resistant strains highlights the need for new therapies, yet regulatory barriers for neglected diseases prolong development timelines. ^57^^,^^63^^–^^65^ Another possible reason for the decline in new studies after this period is the shift toward computational drug discovery, particularly in silico models, which offer cost-efficient strategies for identifying drug targets. ^66^ However, translating these computational advances into practical treatments for CD remains challenging. ^67^ Together, these factors create a complex and difficult environment for developing novel CD therapies.

A persistent lack of funding dedicated to CD research and treatment development is a significant obstacle to therapeutic progress. ^58^^–^^60^ The lack of financial resources limits researchers’ capacity to conduct robust clinical trials and acquire essential resources. Compounding this issue, the pharmaceutical industry shows limited interest due to the low commercial potential of medications targeting diseases prevalent in low-income regions. ^61^^,^^62^ Furthermore, the emergence of drug-resistant strains emphasizes the urgency of finding alternative treatments, yet regulatory hurdles associated with approving novel therapeutics for neglected diseases further contribute to delays. ^57^^,^^63^^–^^65^ Collectively, these factors create a complex and discouraging landscape that severely hinders the development of new CD treatments.

In Brazil, researchers conducted three-quarters of the studies that comprise this meta-analysis. This country is a natural hub for study in this field since it is one of the nations most impacted by CD, has a robust research infrastructure, and an epidemiological database. ^68^^,^^69^ Research in the nation has also been enhanced by the presence of academic institutions, centers of excellence in tropical health, and financial and research resources. ^70^ On the other hand, even though other Latin American nations are also impacted by CD, scientific research and the health system face structural and financial obstacles that may hinder study. ^71^ Regarding North American countries like the United States of America and Canada, involvement in CD research may depend on the financing available for global health research initiatives and the interests of certain academics or groups. ^72^^,^^73^ The lack of urgency in researching and developing therapies may be attributed to the disease’s low incidence in this area. ^73^^,^^74^ Only one study from France was considered in the meta-analysis. However, it should be considered that, although non-endemic countries do not directly suffer from CD in their populations, they should consider globalization and human mobility, since these factors increase the possibility of transfer. of diseases to non-endemic countries. ^75^^–^^77^ Beyond the moral need to alleviate animals’ suffering, the worldwide scope of CD necessitates a cooperative, international effort to solve the problems this neglected tropical disease presents.

The systematic review and meta-analysis of preclinical studies on CD treatment highlights the critical need for rigor in experimental designs, emphasizing the high risk of bias observed across the included studies. The assessment using SYRCLE’s tool revealed prevalent issues with selection, performance, and detection biases, notably due to inadequate randomization, blinding procedures, and incomplete reporting, which undermines the reliability of findings. ^78^ ^,^ ^79^ Despite these concerns, the GRADE evaluation presented promising efficacy of treatments, with moderate certainty assigned to several studies. These findings suggest that, while preclinical models indicate potential treatment effectiveness, clinical validation in humans remains essential for confirmation. ^80^ A few studies showed higher certainty due to demonstrated efficacy in animal models, particularly in mice, underscoring the importance of further validation. ^17^ ^,^ ^81^ This review is essential as it identifies both the potential and limitations of current treatment strategies, emphasizing the urgent need for more rigorous and clinically relevant preclinical research to bridge the gap between animal models and human applications in CD therapy.

The results of this study, bis-triazole DO870 and VNI have demonstrated promise as treatments for CD in the acute and chronic stages, respectively. The assessment of these substances’ safety and effectiveness in actual clinical settings is unknown, as no published clinical trials have been performed too far. Similarly, only two of the treatment options included in this meta-analysis—posaconazole and fexinidazole—have undergone clinical trial evaluation. Posaconazole’s clinical research shows that, although exhibiting trypanostatic action during therapy, it was ineffective in treating asymptomatic *T. cruzi* carriers in the long term. By contrast, it was demonstrated that benznidazole monotherapy outperformed posaconazole, with high RT-PCR conversion rates lasting up to a year. However, in 32% of instances, posaconazole side effects resulted in medication termination. ^82^ The various regimens had an acceptable safety profile in the clinical research evaluating fexinidazole; nonetheless, they were ineffective in treating *T. cruzi* infection. This has led to discontinuing fexinidazole monotherapy development as a treatment for *T. cruzi* infection. ^83^ These results highlight the necessity of investigating alternative therapeutic approaches to treat CD as well as the significance of conducting thorough assessments of the safety and effectiveness of medicines in clinical trials.

Both benznidazole and nifurtimox have been demonstrated to be more successful in lowering parasitemia during the acute phase of CD. These drugs work by preventing the parasite from synthesizing its DNA, which prevents it from multiplying and spreading throughout the host. ^84^ This effect is mediated by their activation via parasitic nitroreductases (e.g., TcNTR), which reduce their nitro groups into cytotoxic metabolites. These reactive intermediates form adducts with parasite DNA, proteins, and lipids, disrupting essential metabolic pathways. ^85^ Furthermore, it has been noted that these medications have the ability to cause oxidative damage in the parasite, through the generation of superoxide radicals and peroxynitrite during redox cycling of their nitro groups, which helps to eradicate it. ^86^^,^^87^ Nevertheless, in the chronic stage of CD, only a limited effectiveness of current treatments has been observed. This could be attributed to multiple factors, including the persistence of metabolically quiescent parasite forms in certain tissues, their limited accessibility to drugs, the host’s compromised immune response, and disease-induced tissue damage. ^88^^,^^89^ Consequently, overcoming these challenges, particularly the role of dormant parasite populations in therapeutic failure, has become a critical focus in preclinical research for chronic CD, a phase poorly managed by conventional medications. As was previously indicated, VNI has demonstrated encouraging outcomes for CD’s chronic phase. This compound inhibits the Trypanosomatidae enzyme CYP51, ^90^ which prevents the parasites from synthesizing vital sterols and ultimately compromises the integrity of their cell membranes, killing them. ^91^ It also lessens cardiac fibrosis and inflammation, which may indicate a possibility for stopping or healing heart damage brought on by CD. ^49^ With a wide range of activity against different strains of Trypanosoma infections, including resistant ones, and the potential for improved pharmacokinetics and reduced side effects, ^92^^–^^94^ this class of inhibitor may offer safer and more efficient alternatives for treating CD during its chronic stage.

Within this species, the strains of *Trypanosoma cruzi* exhibit unique genetic variations. Regarding their genome, virulence, medication resistance, and capacity to elude the host immune system, each of these strains is distinct. ^95^^,^^96^ These variations may have an impact on how CD progresses and how affected individuals respond to treatment. ^97^ The main strains of *Trypanosoma cruzi* studied in the meta-analysis were Tulahuen and Y, for the chronic and acute phases, respectively. The Y strain, recognized for its high virulence, is preferably employed in models attempting to reproduce the acute phase of the disease, allowing the evaluation of therapy efficacy in decreasing parasitemia and initial infection symptoms. ^98^ On the other hand, research examining the long-term pathogenesis and progression of the disease, as well as the evaluation of therapeutic interventions targeted at lowering parasitic burden and preventing or reversing associated organic damage, utilize the Tulahuen strain, which is recognized for its adaptability and ability to induce a stable chronic infection. ^99^^,^^100^

Conversely, concerning the animal models utilized to assess novel medications against CD, we may bring up the instance of dogs, who serve as significant parasite reservoirs. ^101^ A canine model that accurately mimics human illness has been developed, which makes it easier to assess novel medicinal agents. Routine effectiveness trials are hampered by the high cost and extended lifespan of dogs. ^102^^,^^103^ Non-human primates have also been considered possible models, although their usage is restricted due to expense, lack of validation, and ethical concerns. ^104^^,^^105^ On the other hand, murine models remain the most popular because of their affordability, portability, and capacity to replicate several facets of human illness. ^17^^,^^106^ As demonstrated by the fact that all of the preclinical investigations in this review were carried out in mouse models, they are therefore invaluable resources for researching *T. cruzi* infection and assessing novel antiparasitic medications.

### Limitations and strengths

The influence on the validity, generalization, and robustness of the results is the intrinsic limitation of the smaller number of scientific publications, ^107^ as is the case in this meta-analysis. A limited data set could not include all available evidence on the topic in question, requiring a cautious interpretation of the results. ^108^ Therefore, it is necessary to support additional research on new treatment options for CD to overcome this deficiency and increase the available scientific evidence. The current meta-analysis enables a thorough assessment of therapy alternatives in a standardized and controlled setting by concentrating on preclinical studies utilizing animal models, laying a strong platform for further research and therapeutic development. Additionally, complex statistical methods, such as those included in the “metafor” package, enable in-depth data analysis, making it easier to control study heterogeneity and accurately estimate treatment effects. ^109^ This helps identify patterns and trends in the effectiveness of treatments, which in turn helps direct decision-making and design of future clinical trials.

Furthermore, it is important to acknowledge that a key limitation of this meta-analysis is its dependence on peripheral parasitemia as the primary efficacy measure for chronic Chagas disease, which may not adequately reflect the disease's complex pathophysiology characterized by tissue-specific parasite persistence and progressive organ damage. ^110^ While this metric provides standardization across preclinical studies, ^111^ its utility is constrained by chronic phase characteristics including low/intermittent parasitemia and poor correlation with tissue parasite burden. ^112^ The SYRCLE tool's identification of reporting inconsistencies for tissue-based endpoints further compounds this limitation. Although compounds like VNI show promising anti-parasitic and immunomodulatory activity, their tissue-level efficacy remains uncertain without standardized assessment of extravascular reservoirs. ^82^ Future preclinical research should incorporate multimodal endpoints including quantitative PCR of tissue parasitic load, histopathological analysis, and functional cardiac assessments to better predict clinical outcomes, while maintaining parasitemia as a practical initial screening parameter.

Another critical limitation of this meta-analysis is the high risk of bias identified across the included studies, which stems from variability in experimental designs, reporting inconsistencies, and non-standardized conditions typical of preclinical research. The overall certainty of the evidence is further compromised by these methodological heterogeneities, limiting the generalizability of the findings. ^113^ While preclinical studies provide valuable initial insights, their translational relevance is constrained by the lack of uniform protocols and the inherent differences between animal models and human pathophysiology. ^114^ These challenges underscore the need for cautious interpretation of the results and highlight the importance of standardizing future preclinical research to enhance the reliability and applicability of the data.

### Implications for future research

To identify potential compounds and therapeutic targets, extensive research, including drug discovery approaches, is necessary. ^115^ Similar to the studies included in this meta-analysis, potential therapeutic options should be evaluated in preclinical models, such as cell cultures and animal disease models, to determine their efficacy and safety. ^116^ Subsequently, substances that have potential in preclinical research may proceed to clinical trials. There are several important reasons why many therapeutic approaches that show promise in preclinical research are never tested in clinical trials. Initially, moving from preclinical research to clinical trials is an expensive and complex procedure that requires meticulous preparation and substantial monetary means. ^117^ Clinical studies to evaluate potential drugs can often be hampered by a lack of money or investment from the pharmaceutical industry or funding bodies. ^118^^,^^119^ Before starting clinical trials, regulatory and safety issues also need to be resolved. Failure to do so could cause further delays or obstacles in transitioning a promising therapy from preclinical to clinical development. ^120^ Additionally, the availability of resources and motivation to conduct clinical trials may be impacted by the lack of interest or priority of the scientific community or the pharmaceutical industry regarding a particular condition, such as CD. ^121^^,^^122^

## Conclusions

This meta-analysis has demonstrated that novel therapeutic options exist that are successful in treating CD; however, most of them are still in the preclinical development stage, and those that have advanced to the clinical trial stage have not demonstrated the best outcomes, meaning that CD treatment remains unresolved. With the primary goal of helping the CD population, the academic community and pharmaceutical companies must collaborate in creating new drugs, as well as continue and apply research that has produced promising results that could hasten the discovery and availability of more effective treatments.

### Ethics and consent

Ethical approval and consent were not required.

## Author contributions

Conceptualization: M.A.C.-P. and M.A.C.-F.; data curation: M.A.C.-P., L.Y.M.-L., B.M.R.-P., and L.D.G.M.; formal analysis: M.A.C.-P. and M.A.C.-F.; funding acquisition: M.A.C.-P., E.A.F.C., and M.A.C.-F.; investigation: L.D.G.M., H.L.B.C., A.S.G., R.A.M.D., R.C.G., and E.A.F.C.; methodology: M.A.C.-P. and M.A.C.-F.; writing—review and editing: L.D.G.M., H.L.B.C., A.S.G., R.A.M.D., R.C.G., and E.A.F.C. All authors have read and agreed to the published version of the manuscript.

## Data Availability

No data are associated with this article. Figshare: Supplementary figure of results from systematic reviews and meta-analyses ( https://doi.org/10.6084/m9.figshare.26240111) ^123^ and Supplementary tables of results from systematic reviews and meta-analyses ( https://doi.org/10.6084/m9.figshare.28635869). ^124^ This project contains the following underlying data:
• Figure S1. A flowchart representing the study selection process’s meta-analysis and systematic review. • Table S1. PRISMA checklist. • Table S2. SYRCLE’s risk of bias tool. • Table S3. GRADE approach. • Table S4. Data results of systematic review of CD treatment possibilities applied to preclinical research with animal model. • Table S5. Data results of meta-analysis of CD treatment possibilities applied to preclinical research with animal model. • Table S6. Reasons for exclusion of full-text reviewed articles. Data are available under the terms of the Creative Commons Attribution 4.0 International license (CC-BY 4.0).
