# Supplementary material for: Treatment options applied to the preclinical studies using animal models for Chagas Disease: a systematic review and meta-analysis
Source: F1000Res. 2025 May 16;13:885. Originally published 2024 Aug 5. [Version 3] doi: 10.12688/f1000research.150723.3 (PMC12123399; doi:10.12688/f1000research.150723.3)
Supplement: Peer review history

# Reviewer report: Reviewer response for version 3

**DOI:** 10.5256/f1000research.182003.r385243

**Published:** 2025-05-28

**Authors:** Juan Carlos Gabaldon

**Affiliations:** 1 Barcelona Institute for Global Health, Universitat de Barcelona, Barcelona, Catalonia, Spain

Authors have adequately addressed my major comments.

Are the rationale for, and objectives of, the Systematic Review clearly stated?

No

Is the statistical analysis and its interpretation appropriate?

Yes

If this is a Living Systematic Review, is the ‘living’ method appropriate and is the search schedule clearly defined and justified? (‘Living Systematic Review’ or a variation of this term should be included in the title.)

No

Are sufficient details of the methods and analysis provided to allow replication by others?

Partly

Are the conclusions drawn adequately supported by the results presented in the review?

Partly

Reviewer Expertise:

Parasitology, tropical medicine, neglected tropical diseases

I confirm that I have read this submission and believe that I have an appropriate level of expertise to confirm that it is of an acceptable scientific standard.

# Reviewer report: Reviewer response for version 2

**DOI:** 10.5256/f1000research.179980.r378071

**Published:** 2025-05-05

**Authors:** Andrea Angheben

**Affiliations:** 1 IRCCS Sacro Cuore Hospital, Negrar, Verona, Italy

I've read author's answers to my review and I recognize that the paper and author's work has been planned with a good methodology but the limits I've highlighted still remain. Although I am not fully convinced that as it stands the paper is well fruible particularly by clinicians and I believe that methods could be improved (particularly with extension of database queries), authors do declare clearly their approach and the paper can be indexed to my opinion.

Are the rationale for, and objectives of, the Systematic Review clearly stated?

Yes

Is the statistical analysis and its interpretation appropriate?

Partly

If this is a Living Systematic Review, is the ‘living’ method appropriate and is the search schedule clearly defined and justified? (‘Living Systematic Review’ or a variation of this term should be included in the title.)

Not applicable

Are sufficient details of the methods and analysis provided to allow replication by others?

Yes

Are the conclusions drawn adequately supported by the results presented in the review?

Yes

Reviewer Expertise:

My area of expertise covers epidemiological and clinical aspects of Chagas disease and other NTDs.

I confirm that I have read this submission and believe that I have an appropriate level of expertise to confirm that it is of an acceptable scientific standard.

# Reviewer report: Reviewer response for version 2

**DOI:** 10.5256/f1000research.179980.r378072

**Published:** 2025-04-26

**Authors:** Juan Carlos Gabaldon

**Affiliations:** 1 Barcelona Institute for Global Health, Universitat de Barcelona, Barcelona, Catalonia, Spain

Authors have adequately addressed most of my comments, and the manuscript has generally improved significantly, particularly from a methodological point of view. I specially appreciated the insightful explanation on why a random effects model was selected despite apparently low heterogeneity, as well as the inclusion of a risk of bias assessment.

 The one significant thing I still believe should be corrected is the interpretation of results during the chronic stage of infection.

 I understand the reasons why authors decided to exclusively evaluate peripheral parasitaemia as an outcome of drug effectiveness in the animal models included in the meta-analysis. However, this is likely inadequate in the chronic stage of disease, when parasitaemia is known to be low and intermittent. Authors indicate that some drugs were found to be effective in the chronic stage of disease, which is also the one of most clinical interest, but such a statement might be inadequate if based exclusively on peripheral parasitaemia. If the effectiveness of compounds in the chronic stage wants to be evaluated, then other outcomes, such as the ones mentioned in my previous review should be included. If only peripheral parasitaemia will be considered, then this should be mentioned and thoroughly discussed in the limitations section.

 Similarly, the discussion section could still be improved. The language in some parts feels artificially complicated, particularly in the first paragraph. On the other hand, the few lines discussing the mechanism of action of BNZ and NFX seem superficial and don’t really describe their effect on parasite nitroreductase, their demonstrated target. There is also no mention on the potential effect of metabolically-quiescent parasite forms on the high rates of therapeutic failure observed in the chronic stage, nor its implications for the design of pre-clinical studies.

 The high risk of bias, and the overall certainty of evidence described by the authors, and their implications for the interpretation of findings should also be discussed in the limitations section, as they severely affect the generalizability of the findings and highlight the difficulties of inferring data exclusively from pre-clinical experiments, which are often carried out in non-standardized conditions.

Are the rationale for, and objectives of, the Systematic Review clearly stated?

No

Is the statistical analysis and its interpretation appropriate?

Yes

If this is a Living Systematic Review, is the ‘living’ method appropriate and is the search schedule clearly defined and justified? (‘Living Systematic Review’ or a variation of this term should be included in the title.)

No

Are sufficient details of the methods and analysis provided to allow replication by others?

Partly

Are the conclusions drawn adequately supported by the results presented in the review?

Partly

Reviewer Expertise:

Parasitology, tropical medicine, neglected tropical diseases

I confirm that I have read this submission and believe that I have an appropriate level of expertise to confirm that it is of an acceptable scientific standard, however I have significant reservations, as outlined above.

# Reviewer report: Reviewer response for version 1

**DOI:** 10.5256/f1000research.165317.r355965

**Published:** 2025-02-13

**Authors:** Andrea Angheben

**Affiliations:** 1 IRCCS Sacro Cuore Hospital, Negrar, Verona, Italy

The paper entitled "Treatment options applied to the preclinical studies using animal models for Chagas Disease: a systematic review and meta-analysis" is an interesting paper exploring the studies on the use of new compounds on animal models of Chagas disease, according to index literature.

 The paper may well interest F1000Research readers. Two compounds seem to be promising but have never reached the clinical phase of evaluation whereas those used on humans have demonstrated not to be superior of existing drugs.

 I have some comments to be addressed before taking a decision on the indexing of the paper. Authors follow a "systematic" process for their literature search but some limitations should be recognized: "only" PubMed has been explored (not Lilacs nor Embase have been explored) and studies with medication added to the well known nifurtimox and benzidazole were not included (why, taking into account control groups?).

 However, methodology for the other aspects is very well described and rigorous, to me. For the statistics, mainly for the graphical representation fo the results, I just ask authors if they can summarize the results of the meta-analysis with diamonds corresponding to the pooled effect of the drugs, or explaining why the prefer not to do it. For a clinician like me, this can be of healp in understanding results very rapidly. 

 Another general comments refers to the facts (also commented by authors) that they identified studies just in a 5 years period and then for around ten years no new studies have been published on animal models. Can this fact be related to a new approach in the field of drugs discovery (in silico models appearance)?. Probably authors should comment also taking into account this aspect in the discussion.

**Finally some minor comments: **in the introduction the phrase "only becoming aware whenever patients are infected even further" is not clear to me; as I understand it seems that CD become clinically evident for continuous exposure to parasites which is not corresponding to the current knowledge. Please clarify. Moreover in the introduction, authors list the difficulties in patient management ending with the phrase "therapeutic failure associated with existing

 therapies". In fact they do not mention and important factor which is also a limitation for the trials in the clinical phase, which is the lack of a cure biomarker and therefor of strong outcomes to be measured when conducting a clinical trial.

Are the rationale for, and objectives of, the Systematic Review clearly stated?

Yes

Is the statistical analysis and its interpretation appropriate?

Partly

If this is a Living Systematic Review, is the ‘living’ method appropriate and is the search schedule clearly defined and justified? (‘Living Systematic Review’ or a variation of this term should be included in the title.)

Not applicable

Are sufficient details of the methods and analysis provided to allow replication by others?

Yes

Are the conclusions drawn adequately supported by the results presented in the review?

Yes

Reviewer Expertise:

My area of expertise covers epidemiological and clinical aspects of Chagas disease and other NTDs.

I confirm that I have read this submission and believe that I have an appropriate level of expertise to confirm that it is of an acceptable scientific standard, however I have significant reservations, as outlined above.

# Response

**Published:** 2025-03-27

**Authors:** Miguel Angel Chavez-Fumagalli

**Affiliations:** Universidad Catolica de Santa Maria, Peru

The paper entitled "Treatment options applied to the preclinical studies using animal models for Chagas Disease: a systematic review and meta-analysis" is an interesting paper exploring the studies on the use of new compounds on animal models of Chagas disease, according to index literature.

 The paper may well interest F1000Research readers. Two compounds seem to be promising but have never reached the clinical phase of evaluation whereas those used on humans have demonstrated not to be superior of existing drugs.

 I have some comments to be addressed before taking a decision on the indexing of the paper. Authors follow a "systematic" process for their literature search but some limitations should be recognized: "only" PubMed has been explored (not Lilacs nor Embase have been explored) and studies with medication added to the well known nifurtimox and benzidazole were not included (why, taking into account control groups?).

**Answer:** Thank you for your valuable and insightful comments. Regarding the exclusive use of PubMed, we selected this database due to its comprehensive and rigorous coverage of peer-reviewed biomedical literature, which aligns with the focus of our review. While we acknowledge that other databases like Lilacs and Embase could provide additional studies, PubMed was sufficient to gather the most relevant and high-quality research for our systematic analysis. In response to your concern about the inclusion of studies involving drug combinations with nifurtimox and benznidazole, it is important to highlight that the exclusion of combination therapies in this systematic review is based on the need to rigorously assess the efficacy of monotherapies in preclinical models of Chagas disease. While clinical data exist on the combination of benznidazole (BNZ) and nifurtimox (NFX), the results of monotherapy are essential to establish a solid comparative baseline and avoid biases from uncontrolled pharmacological interactions. Furthermore, combination therapies present variability in dosage, duration, and potential synergy, which complicates the standardization of results in preclinical studies. By focusing exclusively on monotherapies, we ensure a more accurate evaluation of the individual efficacy of each compound, which is crucial for future therapeutic strategies and the development of new molecules.

 However, methodology for the other aspects is very well described and rigorous, to me. For the statistics, mainly for the graphical representation fo the results, I just ask authors if they can summarize the results of the meta-analysis with diamonds corresponding to the pooled effect of the drugs, or explaining why the prefer not to do it. For a clinician like me, this can be of healp in understanding results very rapidly.

**Answer: **Thank you for your valuable feedback. We appreciate your suggestion regarding the use of diamonds to represent the pooled effect in the graphical representation of the results. However, we chose not to include diamonds in this case due to the specific approach used in our meta-analysis, which was performed using the R programming environment (version 4.2.3) with the "metafor" package. In this package, the pooled effect is typically represented by point estimates and confidence intervals, rather than diamonds. While we understand that diamonds could offer a quick visual summary for clinicians, we prioritized a more precise representation of the data with confidence intervals, which we believe provides a clearer understanding of the variability in the effect sizes.

 Another general comment refers to the facts (also commented by authors) that they identified studies just in a 5 years period and then for around ten years no new studies have been published on animal models. Can this fact be related to a new approach in the field of drugs discovery (in silico models appearance)? Probably authors should comment also taking into account this aspect in the discussion.

**Answer: **We appreciate your comment and thank you for suggesting the inclusion of this aspect. We have added this point to the discussion section.

 Finally, some minor comments: in the introduction the phrase "only becoming aware whenever patients are infected even further" is not clear to me; as I understand it seems that CD become clinically evident for continuous exposure to parasites which is not corresponding to the current knowledge. Please clarify. Moreover, in the introduction, authors list the difficulties in patient management ending with the phrase "therapeutic failure associated with existing therapies". In fact, they do not mention and important factor which is also a limitation for the trials in the clinical phase, which is the lack of a cure biomarker and therefor of strong outcomes to be measured when conducting a clinical trial.

**Answer: **Thank you for your valuable comments. We have revised the manuscript accordingly: the phrase "only becoming aware whenever patients are infected even further" has been clarified to better reflect the current understanding that CD typically becomes clinically evident in the later stages of infection. Additionally, we have addressed the issue of "therapeutic failure associated with existing therapies" by including the important point regarding the lack of a cure biomarker, which limits the ability to measure strong outcomes in clinical trials.

# Reviewer report: Reviewer response for version 1

**DOI:** 10.5256/f1000research.165317.r355966

**Published:** 2025-01-08

**Authors:** Juan Carlos Gabaldon

**Affiliations:** 1 Barcelona Institute for Global Health, Universitat de Barcelona, Barcelona, Catalonia, Spain

The authors provide an interesting work comparing a number of different medications and compounds that have been tested in animal models of Chagas disease and carry out a meta-analysis to summarize the results observed. While I congratulate the author team for the effort put in this well-written work, and for highlighting an important issue, such as the limited availability of medications currently available for the treatment of Chagas disease, I feel that the objective and research question that they intend to answer are unclear, and there are some methodological limitations that prevent me from accepting the document in its current state.

**Introduction: **The introduction summarizes the epidemiological landscape of Chagas disease adequately. However, the idea that BNZ and NFX are ineffective in the chronic stage of disease could soon be reconsidered, and I recommend reading the recent meta-analysis by Rassi et al (Ref 3) showing improved clinical outcomes upon parasitic treatment. It is also increasingly accepted that treatment of infected patients should be the norm, unless specific counter-indications exist. The authors could, in turn, mention that existing therapies still fail to clear parasitaemia in a substantial number of patients, and that since sterilizing cure remains the main objective of therapy, interest in the development of new medications remains justified.

**Methods: **Authors might want to register the systematic review in the PROSPERO database as well, which includes systematic reviews of human and animal studies. Authors should also include the search strategy used in the different datasets as a supplementary file.

 A significant body of Chagas disease literature comes from Latin America, as acknowledged in the review by the large number of works from Brazil, and is sometimes described in Spanish and Portuguese. Considering that the author team seems to have both native Spanish and Portuguese speakers, why were non-English articles excluded from the review and meta-analysis?

 Was blood parasitaemia the only outcome considered by the authors, how was it measured in the different studies, did all of them use PCR, microscopy, was this considered in the inclusion/exclusion criteria?

 An important advantage of animal studies is that they allow a more comprehensive study of the presence of parasites in tissues, such as the digestive tract, the heart or the CNS. This can be done through biopsies, or non-invasive methods using live luminescence, if parasites expressing a luciferase marker are used. An outcome based on the capacity of drugs to clear parasites from deep organs (rather than only clearing peripheral parasitaemia) should be included, given the intermittent nature of parasitaemia in the chronic stages of *T. cruzi* infection.

 Similarly, I disagree with excluding combination therapies. Extensive clinical data shows that the efficacy and safety profile of BNZ and NFX, while far from ideal, has not been beaten by any monotherapy evaluated, which makes it attractive to evaluate the potential role of combined schemes. This could also be included as a sub-analysis comparing combined vs individual treatment schemes.

 However, my major methodological concerns are related to the fact that the authors do not include any form of risk of bias assessment for any of the studies included in the review, or for the review as a whole. There are currently available tools to evaluate the risk of bias of preclinical studies, I recommend checking the SYRCLE’s risk of bias tool Hooijmans CR et.al., 2014 (Ref 1) , which is consistently used for this purpose. Additionally, there is no assessment of the certainty of the evidence provided by this review, which could be obtained by applying the widely used GRADE criteria, useful for both clinical and preclinical studies. I recommend the authors check reference Romantsik O et.al., 2024 (Ref 2), which provides clear guidelines on how to approach the different segments of a preclinical systematic review. No information on the way potential publication bias was addressed is provided.

 Beyond all of this, I don’t seem to completely understand the objective of the meta-analysis. By comparing animals treated with many different compounds, the research question that the authors seem to be wanting to answer is if anti-parasitic treatment (in general) leads to a higher probability of presenting a negative parasitaemia in animals, which seems to have an obvious answer. If this is not the case, I recommend the authors make the research question more explicit in the methods section.

 In the statistical analysis section, authors repeatedly refer to “participants” or “patients”, even though the study focuses exclusively on preclinical studies carried out in animals. The term “subjects” or “animals” should be used here, and consistently throughout the text, to avoid confusion.

 I don’t entirely understand the decision to use a random-effects model for the meta-analysis even though the reported heterogeneity in the different comparisons is consistently very low (reported as 0%). The authors should provide further justification for this decision.

**Results: **Data extraction sheets of the articles that underwent full-text screening should be provided, along with a table indicating the reason why some of these papers were excluded from the meta-analysis. This could be included as an extra column of the table presented in the supplementary files.

 The comparison for which the RR is calculated is not clearly described in the text. Based on my interpretation of the figures and text, authors are measuring the RR of presenting a negative parasitaemia (TC-) at the end of the study, which would explain the RRs > 1 being associated with more effective treatment (a higher "risk" of showing no parasites in blood). However, this is not disclosed in the text and favors confusion. Similarly, the nomenclature system used by the authors to represent the different compounds tested in each study is very confusing. I recommend including the name of the compound used in each line of the Forest plot to improve readability.

 Again, the benefit of seeing the aggregated effect of many different treatment schemes seems unclear to me, as it is just showing that some animals who received some anti-parasitic treatments cleared parasitaemia.

 Justification for the decision

 Systematic reviews of preclinical studies are complex due to the less stringent standardization of methods compared with clinical trials. These reviews often have the objective of prioritizing compounds to move forward for clinical evaluation, and are of value when a large numbers of compounds are being tested in different studies, which does not seem to be the case here. As the authors rightly point out, some compounds whose efficacy was discarded in clinical trials, such as posaconazaole are included in the meta-analysis, while others that show promise to progress to clinical stages, such as benzoxaboroles and cyanotriazoles are not, casting doubts on the applicability of the results.

 I applaud the authors interest in bringing attention to the limited availability of new therapeutic alternatives for Chagas disease, and invite them to keep working on the subject. However, If the authors just want to provide a high-level image of the compounds currently being evaluated in preclinical stages, perhaps a well-structured scoping literature review is a better format to do so, as would also allow them to describe the benefits and pitfalls of more drugs currently under study.

 If the authors feel that a systematic review of preclinical studies is necessary, or the best way to present their work, then I encourage them to design a concise research question they want to answer, and guarantee that issues such as the risk of bias assessment and the certainty of evidence of the work are adequately addressed, as these are essential for the interpretation of the results.

Are the rationale for, and objectives of, the Systematic Review clearly stated?

No

Is the statistical analysis and its interpretation appropriate?

Yes

If this is a Living Systematic Review, is the ‘living’ method appropriate and is the search schedule clearly defined and justified? (‘Living Systematic Review’ or a variation of this term should be included in the title.)

No

Are sufficient details of the methods and analysis provided to allow replication by others?

Partly

Are the conclusions drawn adequately supported by the results presented in the review?

Partly

Reviewer Expertise:

Parasitology, tropical medicine, neglected tropical diseases

I confirm that I have read this submission and believe that I have an appropriate level of expertise to state that I do not consider it to be of an acceptable scientific standard, for reasons outlined above.

## References

[1] : SYRCLE's risk of bias tool for animal studies. *BMC Med Res Methodol* .2014;14: 10.1186/1471-2288-14-43 43 10.1186/1471-2288-14-43 24667063 PMC4230647

[2] : Value of preclinical systematic reviews and meta-analyses in pediatric research. *Pediatr Res* .2024;96(3) : 10.1038/s41390-024-03197-1 643-653 10.1038/s41390-024-03197-1 38615075 PMC11499280

[3] : Impact of antiparasitic therapy on cardiovascular outcomes in chronic Chagas disease. A systematic review and meta-analysis. *eClinicalMedicine* .2025;79: 10.1016/j.eclinm.2024.102972 10.1016/j.eclinm.2024.102972 PMC1173249939810938

# Response

**Published:** 2025-03-27

**Authors:** Miguel Angel Chavez-Fumagalli

**Affiliations:** Universidad Catolica de Santa Maria, Peru

The authors provide an interesting work comparing a number of different medications and compounds that have been tested in animal models of Chagas disease and carry out a meta-analysis to summarize the results observed. While I congratulate the author team for the effort put in this well-written work, and for highlighting an important issue, such as the limited availability of medications currently available for the treatment of Chagas disease, I feel that the objective and research question that they intend to answer are unclear, and there are some methodological limitations that prevent me from accepting the document in its current state.

 Introduction:

 The introduction summarizes the epidemiological landscape of Chagas disease adequately. However, the idea that BNZ and NFX are ineffective in the chronic stage of disease could soon be reconsidered, and I recommend reading the recent meta-analysis by Rassi et al (Ref 3) showing improved clinical outcomes upon parasitic treatment. It is also increasingly accepted that treatment of infected patients should be the norm, unless specific counter-indications exist. The authors could, in turn, mention that existing therapies still fail to clear parasitaemia in a substantial number of patients, and that since sterilizing cure remains the main objective of therapy, interest in the development of new medications remains justified.

**Answer:** Thank you for your insightful comments and for pointing out the recent findings regarding the efficacy of BNZ and NFX in the chronic stage of Chagas disease. We have carefully revised the introduction to incorporate your suggestions.

 Methods:

 Authors might want to register the systematic review in the PROSPERO database as well, which includes systematic reviews of human and animal studies. Authors should also include the search strategy used in the different datasets as a supplementary file.

**Answer:** Thank you for your valuable comments and suggestions. We would like to clarify that our systematic review is registered in the INPLASY database. The registration details are available in the manuscript. Regarding the search strategies, they were developed based on a bibliometric analysis, which is why they are described as a result in the data sources and study selection section.

 A significant body of Chagas disease literature comes from Latin America, as acknowledged in the review by the large number of works from Brazil, and is sometimes described in Spanish and Portuguese. Considering that the author team seems to have both native Spanish and Portuguese speakers, why were non-English articles excluded from the review and meta-analysis?

**Answer: **Thank you for your comment. The exclusion of articles in Spanish and Portuguese was due to methodological reasons to ensure consistency, replicability, and accessibility in data extraction and analysis. Although the team includes native speakers of these languages, incorporating multiple languages could introduce variability in the interpretation of technical terms and statistical data. Moreover, the most impactful scientific databases predominantly index studies in English, facilitating comparability with other meta-analyses and ensuring that our findings are easily verifiable and reusable.

 Was blood parasitaemia the only outcome considered by the authors, how was it measured in the different studies, did all of them use PCR, microscopy, was this considered in the inclusion/exclusion criteria?

**Answer:** Thank you for your valuable observation. In the data review, we have included a section on **Parasitemia Measurement Methods** **,** where we report that parasitemia was assessed using microscopy, PCR, and blood culture across different studies. The methodology used to measure parasitemia was not a criterion for inclusion or exclusion in our analysis. This decision was based on the need to ensure a comprehensive review of the available data, considering that different studies employ various techniques depending on their resources, study design, and diagnostic capabilities. By not restricting the analysis to a specific measurement method, we aimed to capture a broader range of findings, enhancing the generalizability and applicability of our conclusions.

 An important advantage of animal studies is that they allow a more comprehensive study of the presence of parasites in tissues, such as the digestive tract, the heart or the CNS. This can be done through biopsies, or non-invasive methods using live luminescence, if parasites expressing a luciferase marker are used. An outcome based on the capacity of drugs to clear parasites from deep organs (rather than only clearing peripheral parasitaemia) should be included, given the intermittent nature of parasitaemia in the chronic stages of T. cruzi infection.

**Answer:** Thanks for the observation. The use of parasitemia data alone in this study is based on its relevance as a quantifiable and reproducible indicator of therapeutic efficacy in preclinical models of Chagas disease. Assessing parasitemia reduction allows for a direct measurement of the trypanocidal activity of drugs in peripheral circulation, facilitating comparisons between studies and treatments. Furthermore, since this study is a systematic review and meta-analysis of preclinical treatments in animal models, it focused on standardized data widely reported in the scientific literature, prioritizing studies that documented significant reductions in parasitemia as a criterion for therapeutic success. Although the presence of the parasite in deep tissues is relevant in the chronic phase, obtaining such data requires invasive techniques or specific markers that are not always available or standardized across all analyzed studies. Therefore, the selection of parasitemia as the variable of analysis was based on criteria of data availability, comparability, and methodological robustness within the scope of the study.

 Similarly, I disagree with excluding combination therapies. Extensive clinical data shows that the efficacy and safety profile of BNZ and NFX, while far from ideal, has not been beaten by any monotherapy evaluated, which makes it attractive to evaluate the potential role of combined schemes. This could also be included as a sub-analysis comparing combined vs individual treatment schemes.

**Answer:** Thank you for your observation. However, it is important to emphasize that the exclusion of combination therapies in this systematic review is based on the need to rigorously assess the efficacy of monotherapy treatments in preclinical models of Chagas disease. Although clinical data exist on the combination of benznidazole (BNZ) and nifurtimox (NFX), monotherapy results are essential to establish a solid comparative baseline and avoid biases arising from uncontrolled pharmacological interactions. Additionally, combination treatments exhibit variability in dosage, duration, and potential synergy, making it difficult to standardize results in preclinical studies. By focusing exclusively on monotherapies, we ensure a more precise evaluation of each compound’s individual efficacy, which is essential for future therapeutic strategies and the development of new molecules.

 However, my major methodological concerns are related to the fact that the authors do not include any form of risk of bias assessment for any of the studies included in the review, or for the review as a whole. There are currently available tools to evaluate the risk of bias of preclinical studies, I recommend checking the SYRCLE’s risk of bias tool Hooijmans CR et.al., 2014 (Ref 1) , which is consistently used for this purpose. Additionally, there is no assessment of the certainty of the evidence provided by this review, which could be obtained by applying the widely used GRADE criteria, useful for both clinical and preclinical studies. I recommend the authors check reference Romantsik O et.al., 2024 (Ref 2), which provides clear guidelines on how to approach the different segments of a preclinical systematic review. No information on the way potential publication bias was addressed is provided.

**Answer:**  Thank you for your valuable feedback and for taking the time to review our manuscript. In response to your concern regarding the risk of bias assessment, we have applied SYRCLE’s Risk of Bias Tool to assess the risk of bias for each of the studies included in our review. Additionally, we have incorporated the GRADE criteria to assess the certainty of the evidence, to ensure a robust evaluation of the evidence’s quality and applicability.

 Beyond all of this, I don’t seem to completely understand the objective of the meta-analysis. By comparing animals treated with many different compounds, the research question that the authors seem to be wanting to answer is if anti-parasitic treatment (in general) leads to a higher probability of presenting a negative parasitaemia in animals, which seems to have an obvious answer. If this is not the case, I recommend the authors make the research question more explicit in the methods section.

**Answer:** We appreciate your observation and the opportunity to clarify our objective. Our meta-analysis does not simply aim to confirm that antiparasitic treatment reduces parasitemia but rather to compare the efficacy of different compounds in preclinical models of Chagas disease, identifying those with the highest therapeutic potential. Since few treatments have successfully progressed to clinical trials, our study provides a critical evaluation of emerging options and their differences in efficacy depending on the experimental model and disease phase. For the sake of clarity, we will reinforce the explicit formulation of our objective.

 In the statistical analysis section, authors repeatedly refer to “participants” or “patients”, even though the study focuses exclusively on preclinical studies carried out in animals. The term “subjects” or “animals” should be used here, and consistently throughout the text, to avoid confusion.

**Answer: **Thank you for your valuable observation. We acknowledge the inconsistency in terminology in the statistical analysis section and throughout the text. We will replace “participants” and “patients” with “animals”, to ensure clarity and accuracy.

 I don’t entirely understand the decision to use a random-effects model for the meta-analysis even though the reported heterogeneity in the different comparisons is consistently very low (reported as 0%). The authors should provide further justification for this decision.

**Answer: **Thank you for your valuable observation. We appreciate your careful review of our methodological choices. We will provide a clearer justification for the use of the random-effects model in the meta-analysis, ensuring that our decision is well-supported despite the low reported heterogeneity.

 Results:

 Data extraction sheets of the articles that underwent full-text screening should be provided, along with a table indicating the reason why some of these papers were excluded from the meta-analysis. This could be included as an extra column of the table presented in the supplementary files.

**Answer: **Thank you for your valuable comment. We have followed your recommendation and included the data extraction sheets for the articles that underwent full-text screening, along with an additional column in the table in the supplementary files to indicate the reasons for the exclusion of certain papers from the meta-analysis. We appreciate your feedback and believe it improves the clarity of the manuscript.

 The comparison for which the RR is calculated is not clearly described in the text. Based on my interpretation of the figures and text, authors are measuring the RR of presenting a negative parasitaemia (TC-) at the end of the study, which would explain the RRs > 1 being associated with more effective treatment (a higher "risk" of showing no parasites in blood). However, this is not disclosed in the text and favors confusion.

**Answer: **We appreciate your observation regarding the clarity of the RR calculation. We have revised the text to explicitly describe the comparison for which the RR is calculated, ensuring clearer interpretation and avoiding potential confusion.

 Similarly, the nomenclature system used by the authors to represent the different compounds tested in each study is very confusing. I recommend including the name of the compound used in each line of the Forest plot to improve readability.

**Answer:** Thank you for your valuable feedback. We have made the necessary changes and now include the name of each compound used in each line of the Forest plot to improve readability and clarity.

 Again, the benefit of seeing the aggregated effect of many different treatment schemes seems unclear to me, as it is just showing that some animals who received some anti-parasitic treatments cleared parasitaemia.

**Answer: **The meta-analysis conducted in this study provides a quantitative assessment of the impact of various therapeutic options in animal models of Chagas disease, offering a comprehensive view of their preclinical efficacy. While it is true that some treated animals achieved parasitemia clearance, the value of our study lies in the systematic identification of efficacy patterns across different compounds and the comparative evaluation of their relative performance in different disease phases. Rather than merely confirming that some treatments can reduce parasitemia, this analysis allows us to discern which treatments demonstrate greater consistency and potential for future clinical research, helping to focus efforts on the most promising options for the development of new therapies​.

 Justification for the decision

 Systematic reviews of preclinical studies are complex due to the less stringent standardization of methods compared with clinical trials. These reviews often have the objective of prioritizing compounds to move forward for clinical evaluation, and are of value when a large numbers of compounds are being tested in different studies, which does not seem to be the case here. As the authors rightly point out, some compounds whose efficacy was discarded in clinical trials, such as posaconazaole are included in the meta-analysis, while others that show promise to progress to clinical stages, such as benzoxaboroles and cyanotriazoles are not, casting doubts on the applicability of the results.

**Answer: **We appreciate the reviewer’s observation regarding the complexity of systematic reviews of preclinical studies due to methodological variability compared to clinical trials. While many such reviews aim to prioritize compounds for clinical evaluation, our study also serves the critical purpose of identifying therapeutic response patterns and providing a quantitative assessment of treatment efficacy in animal models, which is particularly relevant for neglected diseases like Chagas. The inclusion of posaconazole, despite its unsatisfactory clinical outcomes, remains valuable for understanding its preclinical performance and benchmarking emerging therapies, while the absence of benzoxaboroles and cyanotriazoles was not due to bias but rather the lack of published preclinical studies meeting our inclusion criteria. Our systematic approach, following PRISMA guidelines and registered in INPLASY (INPLASY202430101), ensures transparency and reproducibility, making our findings a valuable contribution to guiding future research and identifying promising candidates for further investigation.

 I applaud the authors interest in bringing attention to the limited availability of new therapeutic alternatives for Chagas disease, and invite them to keep working on the subject. However, If the authors just want to provide a high-level image of the compounds currently being evaluated in preclinical stages, perhaps a well-structured scoping literature review is a better format to do so, as would also allow them to describe the benefits and pitfalls of more drugs currently under study.

**Answer: **We sincerely appreciate the reviewer’s thoughtful observation and encouragement regarding our work on Chagas disease therapeutics. While a well-structured scoping review could indeed provide a broad overview of compounds under preclinical evaluation, our decision to conduct a systematic review and meta-analysis was driven by the need to quantitatively assess treatment efficacy across multiple studies using standardized methodologies. By adhering to PRISMA guidelines, our study synthesizes available preclinical data, providing statistical insights into parasitemia reduction and the therapeutic potential of different compounds. This quantitative approach not only allows for a more rigorous comparison of treatment effects but also highlights trends, gaps, and inconsistencies in the current literature, which are critical for guiding future research and clinical translation. We believe that this level of analysis is essential for evidence-based decision-making and prioritization of drug candidates for Chagas disease treatment. Nonetheless, we acknowledge the value of a scoping review in complementing our findings by exploring a broader range of therapeutic strategies beyond efficacy metrics, and we appreciate the suggestion for future research directions.

 If the authors feel that a systematic review of preclinical studies is necessary, or the best way to present their work, then I encourage them to design a concise research question they want to answer, and guarantee that issues such as the risk of bias assessment and the certainty of evidence of the work are adequately addressed, as these are essential for the interpretation of the results.

**Answer:  **Thank you for your valuable comments and suggestions. In response to your observation, we have made the necessary changes to the manuscript. We have refined the research question to ensure it is clear and concise, accurately reflecting the study's objective. Additionally, we have conducted a risk of bias assessment using the SYRCLE tool and addressed the certainty of the evidence using the GRADE approach, as you recommended. These improvements enhance the robustness of our analysis and ensure a more reliable interpretation of the results.
